# Mirror proteorhodopsins

**DOI:** 10.1038/s42004-023-00884-8

**Published:** 2023-05-02

**Authors:** Ivan S. Okhrimenko, Kirill Kovalev, Lada E. Petrovskaya, Nikolay S. Ilyinsky, Alexey A. Alekseev, Egor Marin, Tatyana I. Rokitskaya, Yuri N. Antonenko, Sergey A. Siletsky, Petr A. Popov, Yuliya A. Zagryadskaya, Dmytro V. Soloviov, Igor V. Chizhov, Dmitrii V. Zabelskii, Yury L. Ryzhykau, Alexey V. Vlasov, Alexander I. Kuklin, Andrey O. Bogorodskiy, Anatolii E. Mikhailov, Daniil V. Sidorov, Siarhei Bukhalovich, Fedor Tsybrov, Sergey Bukhdruker, Anastasiia D. Vlasova, Valentin I. Borshchevskiy, Dmitry A. Dolgikh, Mikhail P. Kirpichnikov, Ernst Bamberg, Valentin I. Gordeliy

**Affiliations:** 1 Research Center for Molecular Mechanisms of Aging and Age-related Diseases, Moscow Institute of Physics and Technology, Dolgoprudny, Russia; 2 European Molecular Biology Laboratory, Hamburg, Germany; 3 Shemyakin–Ovchinnikov Institute of Bioorganic Chemistry, RAS, Moscow, Russia; 4 Belozersky Institute of Physico-Chemical Biology, Lomonosov Moscow State University, Moscow, Russia; 5 iMolecule, Skolkovo Institute of Science and Technology, Moscow, Russia; 6 Institute for Biophysical Chemistry, Hannover Medical School, Hannover, Germany; 7 European XFEL, Schenefeld, Germany; 8 Frank Laboratory of Neutron Physics, Joint Institute for Nuclear Research, Dubna, Russia; 9 Biological Faculty, Lomonosov Moscow State University, Moscow, Russia; 10 Max Planck Institute of Biophysics, Frankfurt am Main, Germany; 11 Institut de Biologie Structurale (IBS), Université Grenoble Alpes, CNRS, CEA, Grenoble, France; 12 Present Address: Groningen Biomolecular Sciences and Biotechnology Institute, University of Groningen, Groningen, The Netherlands

**Keywords:** X-ray crystallography, Ion channels, Membrane proteins, Biophysical chemistry, Transporters

## Abstract

Proteorhodopsins (PRs), bacterial light-driven outward proton pumps comprise the first discovered and largest family of rhodopsins, they play a significant role in life on the Earth. A big remaining mystery was that up-to-date there was no described bacterial rhodopsins pumping protons at acidic pH despite the fact that bacteria live in different pH environment. Here we describe conceptually new bacterial rhodopsins which are operating as outward proton pumps at acidic pH. A comprehensive function-structure study of a representative of a new clade of proton pumping rhodopsins which we name “mirror proteorhodopsins”, from *Sphingomonas paucimobilis* (*Spa*R) shows cavity/gate architecture of the proton translocation pathway rather resembling channelrhodopsins than the known rhodopsin proton pumps. Another unique property of mirror proteorhodopsins is that proton pumping is inhibited by a millimolar concentration of zinc. We also show that mirror proteorhodopsins are extensively represented in opportunistic multidrug resistant human pathogens, plant growth-promoting and zinc solubilizing bacteria. They may be of optogenetic interest.

## Abstract (web summary)

Proteorhodopsins act as light-driven outward proton pumps mainly at neutral and alkaline pH, however, mirror proteorhodopsins are known to operate only at acidic pH. Here, the authors report the detailed structural and functional characterization of pH mirror proteorhodopsin SpaR as well as its diverse biological roles.

## Introduction

The discovery of microbial rhodopsins in bacteria in 2000 by Béjà et al.^1^ started the era of proteorhodopsins, outward light-driven proton pumps, the largest family of rhodopsins. These proteins comprise DTD, DTE or DTK motifs at the places of D85 (proton acceptor), T89 and D96 (proton donor) of bacteriorhodopsin from *Halobacterium salinarum* (BR)^2^ with the corresponding DTD motif. As we have mentioned, in contrast to archaeal and eukaryotic outward proton pumps, which pump protons in a wide range of pH, proteorhodopsins translocate protons only at neutral and alkaline pH^2,3^.

A new phylogenetic group of rhodopsins with DTG motif was identified by Harris et al.^4^ The authors reported proteorhodopsins derived from *Pseudomonas putida (Psp*R*)* and *Pantoea ananatis (Pa*R*)*, in which the carboxylic proton donor to the retinal Schiff base (RSB) (D96 in BR) is replaced by G84. In addition, the hydrogen-bonding partner T46 of D96 in BR is replaced by histidine, which is also conserved in the whole DTG group (Supplementary Figs. 1, 2). The authors showed proton-pumping ability of rhodopsins. However, since the corresponding experiments were done with the proteins in unbuffered solutions the authors did not reveal pH dependence of the proton pumping. Recently Cho et al. claimed that *Mp*R, another representative of DTG clade *Methylobacterium populi*, pumps Li^+^ and Na^+^ ions^5^ protons. Thus, the functional properties of rhodopsins with a DTG motif have not been well understood.

Moreover, recently we identified a new clade of rhodopsins characterized by a DTS motif^4,6,7^ and showed that a representative of the clade, *Spa*R rhodopsin from an opportunistic *Sphingomonas paucimobilis*, operates as an outward proton pump at acidic but not at neutral and alkaline pH^3,8^. DTS and DTG motifs are quite close and this fact strongly motivates additionally to perform a comprehensive study of DTS/DTG rhodopsins (Supplementary Figs. 2, 3).

An intriguing fact, not recognized previously, is that all the mentioned above partially characterized rhodopsins originate from opportunistic pathogens (*Pseudomonas putida*^9^ *(Psp*R^4^*), Pantoea ananatis*^10,11^ *(Pa*R^4^*), Sphingomonas paucimobilis*^12^ and *Methylobacterium populi*^13^*)*.

Our analysis of the existing literature data on the bacteria show another common exciting property - they are zinc resistant^11,13–16^. It is known that zinc plays an important role in pathogen bacteria-host interactions^17–19^. Pathogenic bacteria could exploit the interaction with Zn^2+^ because Zn^2+^ role in organism is difficult to overestimate^20^. Zn^2+^ is an essential part of the immune system of mammalians, in particular in action against bacterial infections^18,21,22^. Zn^2+^ affects multiple aspects of immunity - both innate and adaptive^23^, Zn^2+^ deficiency in aging is involved in the shift of immune cells balance^24^.

An important question is whether it is a coincidence or DTS(G) motifs do support an important biological function in the bacteria. If yes, then what is this biological function? Is this function common to all DTS and DTG rhodopsins? Are they all pumping protons at acidic but not at neutral and alkaline pH values? How big is the family of these proteorhodopsins? What is their role in the infections? Lack of functional and structural data, as-yet unclear mechanism of the function of DTG and DTS rhodopsins did not allow answering these questions.

**Fig. 1:**
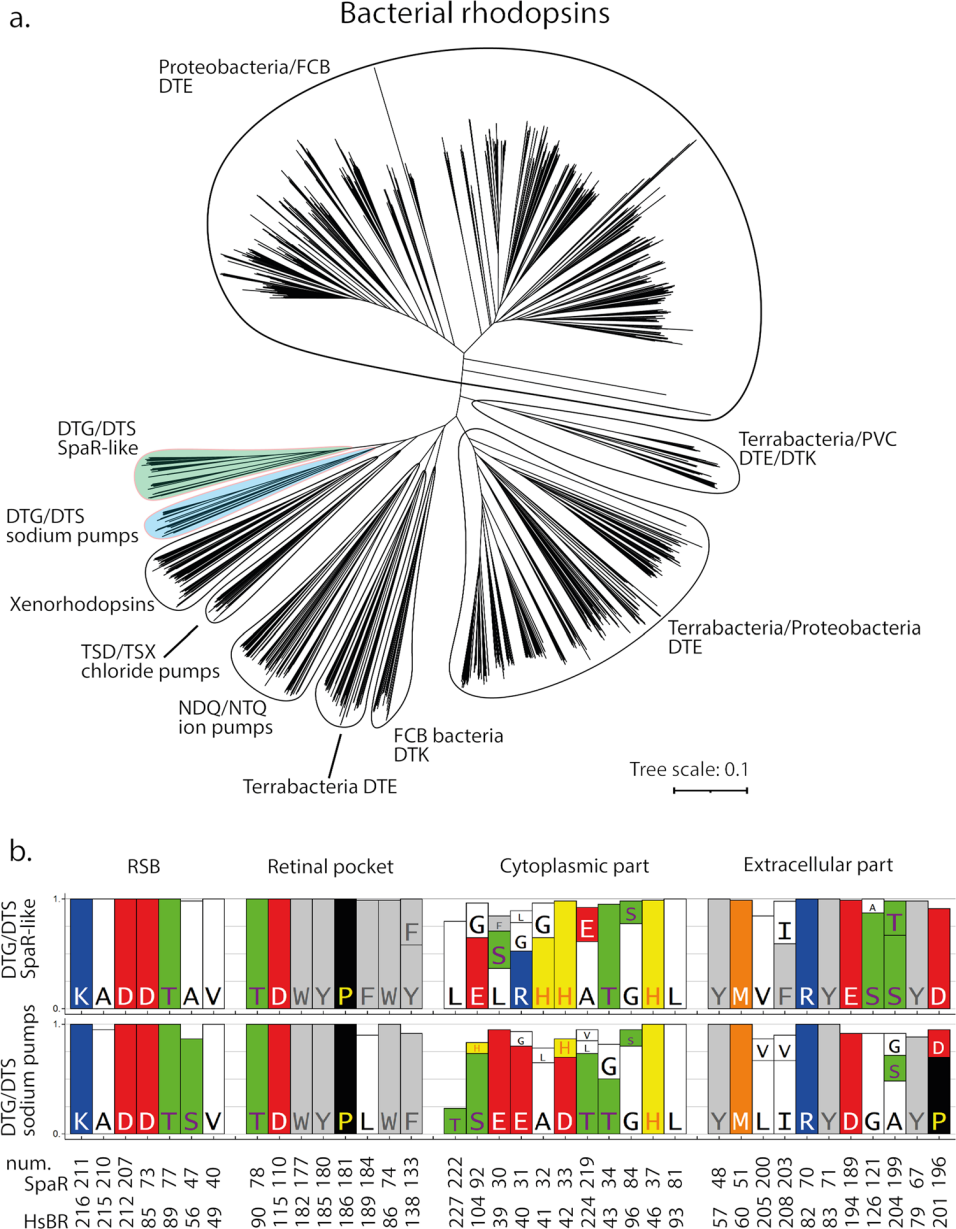
Phylogenetic analysis of bacterial rhodopsins. **a** Phylogenetic tree of bacterial rhodopsins. The clades which consist mostly of DTG/DTS motif rhodopsins are highlighted in colour: green – *Spa*R-like proteins, blue – *Mp*R-like proteins (sodium pumps). **b** Comparison of the conservative amino acid patterns of *Spa*R-like and *Mp*R-like rhodopsins.

First, in our work we used bioinformatics to search for *Spa*R-like rhodopsins and found a large clade of such rhodopsins showing that *Spa*R is a representative of a distinct clade of proteorhodopsins with unique properties (Fig. 1). We name this family as ‘mirror proteorhodopsins (mPRs)’ and argue that they may play a distinguishing role among rhodopsins. We showed that most of the proteins with a DTG motif, including those found in^4^ also belong to the clade. However, some of rhodopsins with a DTG motif (including a sodium pump described in ref. ^5^) belong to a separate clade. Here we report a comprehensive structure-function study of *Spa*R from genome of strictly aerobic bacteria *Sphingomonas paucimobilis*^25^, isolated from hospital ventilation and found in a range of water and land habitats, and clinical samples^12^. We confirmed that *Spa*R operates as a light-driven proton pump at pH < 6.5. The crystal structure of *Spa*R at 2.8 Å resolution is markedly different from those of the known light-driven proton pumps and, unexpectedly, has remarkable similarities to channelrhodopsins. This predetermines the *Spa*R unique functional properties. We showed that *Spa*R properties are zinc dependent. Moreover, at mM concentrations of Zn^2+^, outward proton pumping is inhibited. We also demonstrated that *Spa*R can be expressed in lysosomes of animal cells and the pH of selectively acid lysosomes can be controlled with light. It means that *Spa*R can be potentially used in optogenetics to selectively control pH of the cells and their organelles at acidic conditions.

We found mirror proteorhodopsins genes in genomes of some multidrug-resistant bacteria known as opportunistic human pathogens and/or are involved in plant infections. The identified mPRs mainly belong to alpha- and gamma-Proteobacteria, first of all to *Sphingomonas* and *Pseudomonas* (Supplementary Fig. 4). This large rhodopsin clade deserves a special attention. Our analysis also confirms that the new clade sodium pumping DTG/DTS rhodopsins is a separate and is significantly different from sodium pumping rhodopins with an NDQ motif ^26–28^ and therefore will also attract attention.

In this work we present a detailed biophysical characterization of a new microbial rhodopsin of the clade ‘Mirror proteorhodopsins‘ with the unique properties. This name suggests function at acidic pH (below pH 6.5) inversely to the well described proteorhodopsins which translocate protons at neutral and alkaline pH. The rhodopsin of *Sphingomonas paucimobilis* named *Spa*R has structure more similar to ChR family than to proteorhodopsins. The histidine residues at the ChR-like cavities and gates explain the inhibition of the light-driven proton-pumping activity by zinc ions.

## Results

### Identification of a new distinct family of rhodopsins

Previously, we found a rhodopsin in *Sphingomonas paucimobilis (Spa*R*)* with a DTS motif ^6,8^. To understand how many rhodopsins share similarities to *Spa*R, we bioinformatically retrieved all rhodopsin genes of bacteria from the UniParc database. We should note that the DTS motif was also found in rhodopsins of other clades, including bacterial: of the viral group-I rhodopsin present in Choano Virus, in PgV^7^ and xenorhodopsin clade (e.g., Anabaena sensory rhodopsin, ASR^29–31^). This means that a the three-letter motif is insufficient to predict rhodopsin functions. Thus, we clustered them into subgroups based on their similarity to each other (blastp e-value threshold of 1e-65) and retrieved a clade of *Spa*R-like rhodopsins. The phylogenetic tree of bacterial rhodopsins shows *Spa*R-like rhodopsins form a distinct clade, which consists of 103 rhodopsins with DTG (including DTG-rhodopsins from Harris et al.^4^), DTS (including *Spa*R^6,8^ and DTT proteins (Fig. 1a). Given a very high sequence similarity within the group, we presume that all its members function similarly.

Interestingly, quite a considerable number of 60 rhodopsins with DTG and DTS motifs belong to a separate clade (the clade consists of 48 DTG, 9 DTS, 2 DTA, and 1 DTT proteins). One of the representatives of this clade with a DTG motif was recently reported to be a Na^+^/Li^+^ light-driven rhodopsin (*Mp*R) from *Methylobacterium populi*^5^. This finding was unexpected since another DTG motif rhodopsin characterized by Harris et al.^4^ displayed proton pumping. We performed an in-depth analysis of the sequences to resolve this apparent contradiction. Indeed, two clades differ from each other in terms of conservative amino acid patterns (Fig. 1b). Whereas *Spa*R-like subfamily possesses highly conservative H32, H33 (*Spa*R numbering) at the cytoplasmic part of the protein, most of *Mp*R-like DTG/DTS motif rhodopsins^5^ share carboxylic amino acids E32, E33, and D35 (*Mp*R numbering, corresponds to 30, 31, and 33 in *Spa*R) in the similar region. As for the extracellular side of the proteins, S121 and S199 (*Spa*R numbering) are found in a vast majority of *Spa*R-like rhodopsins, but not in *Mp*R-like proteins.

To understand better *Spa*R-like rhodopsins, we performed a comprehensive functional and structural characterization of a representative of the clade - *Sphingomonas paucimobilis rhodopsin (Spa*R*)*. The study supports the idea that the clade is functionally and structurally unique.

### Functional characterization of *Spa*R

The *Spa*R gene was expressed in *E. coli* as previously described in ref. ^5^. The size-exclusion chromatography (SEC) of *Spa*R at five different pH values (4.4, 5.4, 6.4, 7.4, 8.4) showed that the dominating oligomer of the protein is trimer in a wide range of pH (Supplementary Fig. 5). At pH 4.4 only a single peak corresponding to a trimeric form of the protein is observed. Additional peaks corresponding to higher molecular weights appear with an increase of pH.

Small-angle X-ray scattering (SAXS) studies of solubilized *Spa*R also suggest trimeric organization of rhodopsins. The small-angle part of the scattering curves indicates some aggregation of the protein. We used the range of the scattering vectors *q* > 0.04 Å^−1^ to eliminate from our data analysis the scattering from the aggregates^32^. The experimental SAXS profile at pH 7.2 in a range of 0.04 Å^−1^ < q < 0.26 Å^−1^ was fitted using MEMPROT^33^. The data show that the trimer of SpaR fits the SAXS data, whereas the monomer does not (Supplementary Fig. 6). The same trimers are also observed in the crystal packing of the protein described below. Taken together, we believe that the trimer is the native oligomerization state of the protein.

**Fig. 2:**
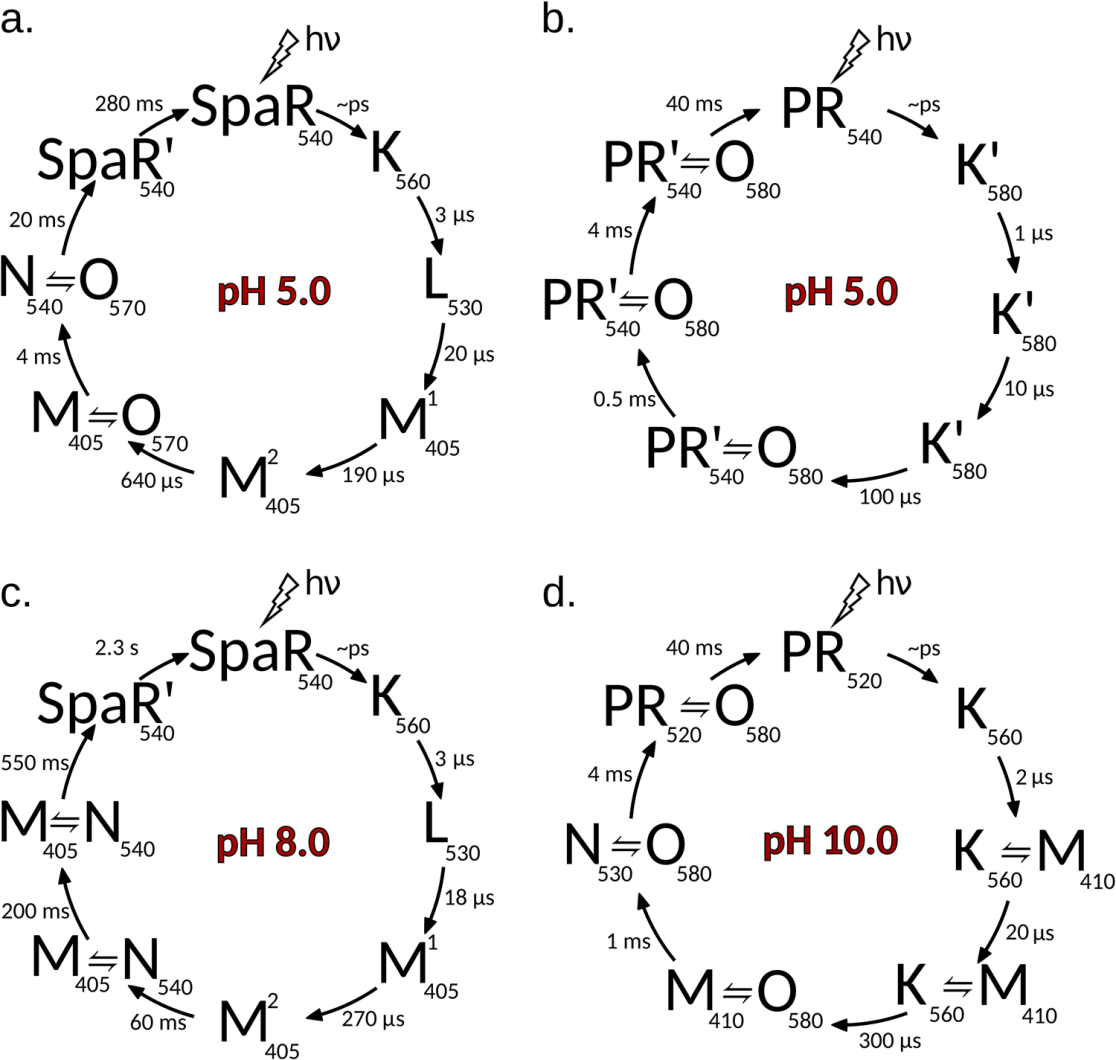
Photocycles of solubilized rhodopsins at acidic and alkaline pH. Photocycle of SpaR at (**a**) pH5.0, at (**b**) pH8.0. Photocycle of green proteorhodopsin (PR)^3^ at (**c**) pH5.0, at (**d**) pH10.0.

The absorption maximum of retinal Schiff base (RSB) of DDM-solubilized *Spa*R corresponds to 540 nm at pH 7.5, and does not depend on pH in a range from 2.5 to 11. At pH lower than 2.5 maximum of the absorption spectra is shifted due to the titration of proton acceptor group D73 (corresponds to D85 in *H. salinarum* bacteriorhodopsin). The pK of proton acceptor group of SpaR was estimated as ~1.0 (Supplementary Fig. 7) which is much lower than in known proteorhodopsins^2,3^. The photocycle of *Spa*R was determined by flash-photolysis as described in Methods (Supplementary Fig. 8). The duration of the photocycle depends dramatically on pH (Fig. 2a, c; Supplementary Figs. 9, 10). The time-constants of the decays of the intermediate states were obtained by the global seven-exponential fitting of the multi-wavelength kinetics at pH from 4.6 to 8.0. The influence of protonation of the presumable proton donor group (corresponds to D96 in *H. salinarum* bacteriorhodopsin) on the apparent half-time of the Schiff base re-protonation was described and *pK* of proton donor group was estimated as 5.8 ± 0.2 using single exponential approximation of the transient absorption decay at 400 nm at different pH (Supplementary Fig. 10, similar way was estimated for ESR^34^). Using BR and green proteorhodopsin (PR) photocycles as references^35,36^, we conclude that the photocycle of *Spa*R comprises the following intermediates: K, L, M_1_, M_2_, N_1_, N_2_, and N_3_-like. At pH 5.0, the entire photocycle lasts about 90 ms (Fig. 2a, Supplementary Fig. 8). At pH 8.0, the photocycle is much slower −2.3 s (Fig. 2c). In addition, in opposite to pH 5.0, the N_3_ phase is absent at pH 8.0 (Fig. 2c, Supplementary Fig. 8).

The dramatic increase of the photocycle duration of *Spa*R at higher pH is not inherent to BR or proteorhodopsins, though proteorhodopsins proton transport properties do depend on pH (Fig. 2b, d). It was reported that PR has acidic and alkaline forms of D97, which serves as the primary acceptor of the RSB proton under alkaline conditions but is protonated under acidic conditions^3^. As a result, the M_2_-state is not accumulated in the acidic form (Fig. 2b).

The changes of pH of the *E. coli* cells suspension with expressed SpaR in the 100 mM NaCl solution upon light illumination are shown in Supplementary Fig. 11b. The observed effect of lowering pH points out to proton translocation outward the cell since it was considerably reduced in the presence of the protonophore carbonyl cyanide m-chlorophenyl hydrazone (CCCP). Notably, the effect was not completely abolished by CCCP. We performed similar experiments with *Spa*R reconstituted into lipid vesicles. In this case, at acidic pH *Spa*R also produced light-induced acidification, confirming the ability of the protein to pump protons, but no effect was observed at pH 7.5 (Supplementary Fig. 11a). These results are consistent with strong pH dependence of the *Spa*R photocycle.

The pH-‘mirror’ behavior of *Spa*R is unusual in comparison with the known proteorhodopsins. Indeed, proteorhodopsins functioning is also pH dependent as they operate as outward proton pumps only at acidic pH. The protonation state of PR D97 (pKa_D97_ = 7.68^3^) is crucial for this variable vectorality. However, *Spa*R shows completely different pH dependence, operating only as an outwardly directed proton pump only at acidic pH and having no pumping activity under normal and alkaline conditions.

### Functional studies of SpaR proteoliposomes

We checked the assembly of the unilamellar structure of proteoliposomes consisting of soybean lecithin phospholipids by using the SAXS option of the Rigaku X-ray station (see Methods). The data (Supplementary Fig. 12a) were fitted by the model of the unilamellar lipid vesicles (ULV) with a polydispersity, χ^2^ = 1.1 and 1.4 for pure liposomes and proteoliposomes, respectively. Different pair-distance distribution functions P(r) for pure liposomes and proteoliposomes (Supplementary Fig. 12b) indicate different size distribution, which is also confirmed by the polydispersity parameters obtained by the fit (*R* = 590, σ/R = 0.34 and R = 310, σ/R = 0.36 for pure liposomes and proteoliposomes, respectively). Despite the different size distribution, the SAXS data confirms the assembly of the liposomes and their unilamellar structure for pure liposomes as well as for proteoliposomes. Guinier-approximation for flat particles ln(I(q)*q^2^) *vs* q^2^ (Supplementary Fig. 12c) results in *R*_*t*_ from 31.2 ± 2.2 Å in the case of pure liposomes to 34.2 ± 1.0 Å for proteoliposomes. The change of the parameter *R*_*t*_ for liposomes after the incorporation of the proteins indicates some changes in overall bilayer structure. The electron density of the protein (~0.42 e/A^3^) is more than for lipid hydrophobic tails (usually < 0.3 e/A^3^); therefore, the reconstitution of the transmembrane proteins into a bilayer leads to an increase of average electron density in its hydrophobic part. This effect can be observed when comparing the profiles of the contrast (*Δρ(z)* = *ρ(z) - ρ*_*buf*_) of electron density (Supplementary Fig. 12d, data presented in relative units) calculated by fitting the SAXS data, which also confirms the reconstitution of the protein into liposomes.

To learn more about the movement of the charges inside the protein along the photocycle, we performed time-resolved studies of the electrogenic behavior of the protein reconstituted in proteoliposomes. The generation of transmembrane electric potential ΔΨ in response to a laser flash illumination of *Spa*R proteoliposomes was observed (Supplementary Fig. 13). The rise of the membrane potential corresponds to the transfer of the positive charge through the membrane. The rise of ΔΨ is the same as for BR^37^ suggesting the pumping of protons inside liposomes (or outward the cells, respectively). We resolved four phases of the potential increase kinetics at pH 7.5 (Supplementary Fig. 13a, b): 0.012 ms (20% of amplitude), 0.16 ms (6%), 13.1 ms (14%), and 57.8 ms (60%). Finally, we observed that the membrane potential was dissipating as it is expected due to the secondary passive leak of ions through the membrane in the time scale of several seconds (Supplementary Fig. 13b). The 0.012 ms component reflects the electrogenic transfer of a proton from the RSB to primary acceptor during M_1_ formation. The 0.16 ms phase corresponds to the rise of the M_2_ state. Phases of 13 ms and 58 ms (which are altogether 74% in amplitude) reflect probably the proton transfer corresponding to the RSB reprotonation during M_2_-to-N_1_ state transition. The electrogenic steps associated with several hundred millisecond and seconds transitions (observed spectroscopically in the photocycle of *Spa*R) are not resolved in the single-turnover kinetics of membrane potential generation. As it can be concluded from our data, the late stages of the photocycle, including slow part of M-N transitions and the process of RSB reisomerization, are not electrogenic or these stages are rather slow so that the potential generation can be masked due to passive proton leakage through the membrane. For this reason (from the deceleration of the photocycle to the characteristic times of passive leakage/discharge of protons through the membrane at neutral and alkaline pH), the data confirm that at neutral and alkaline pH *Spa*R cannot operate effectively as a proton pump in a multiturnover mode.

The decrease of pH from 7.5 to 5.5 results in faster kinetics of membrane potential generation (Supplementary Fig. 13c). The following phases are resolved at pH 5.5: 0.012 ms (24% of amplitude), 0.22 ms (9%), 2.7 ms (46%), and 6.8 ms (21%). So, the phases of the electrogenic proton transfer corresponding to M_2_-decay are ~15–20 times faster than those at pH 7.5, which significantly exceeds the characteristic times of passive leakage/discharge of protons through the membrane. That is, these data confirm that *Spa*R at acidic pH can operate effectively as a proton pump. It should be noted that the opposite is observed in proteorhodopsins (for example, ESR^34^): at acidic pH values, the ability to pump a proton disappears both in experiments with the generation of a membrane potential in a single photocycle mode and in multi-turn measurements^38,39^.

**Fig. 3:**
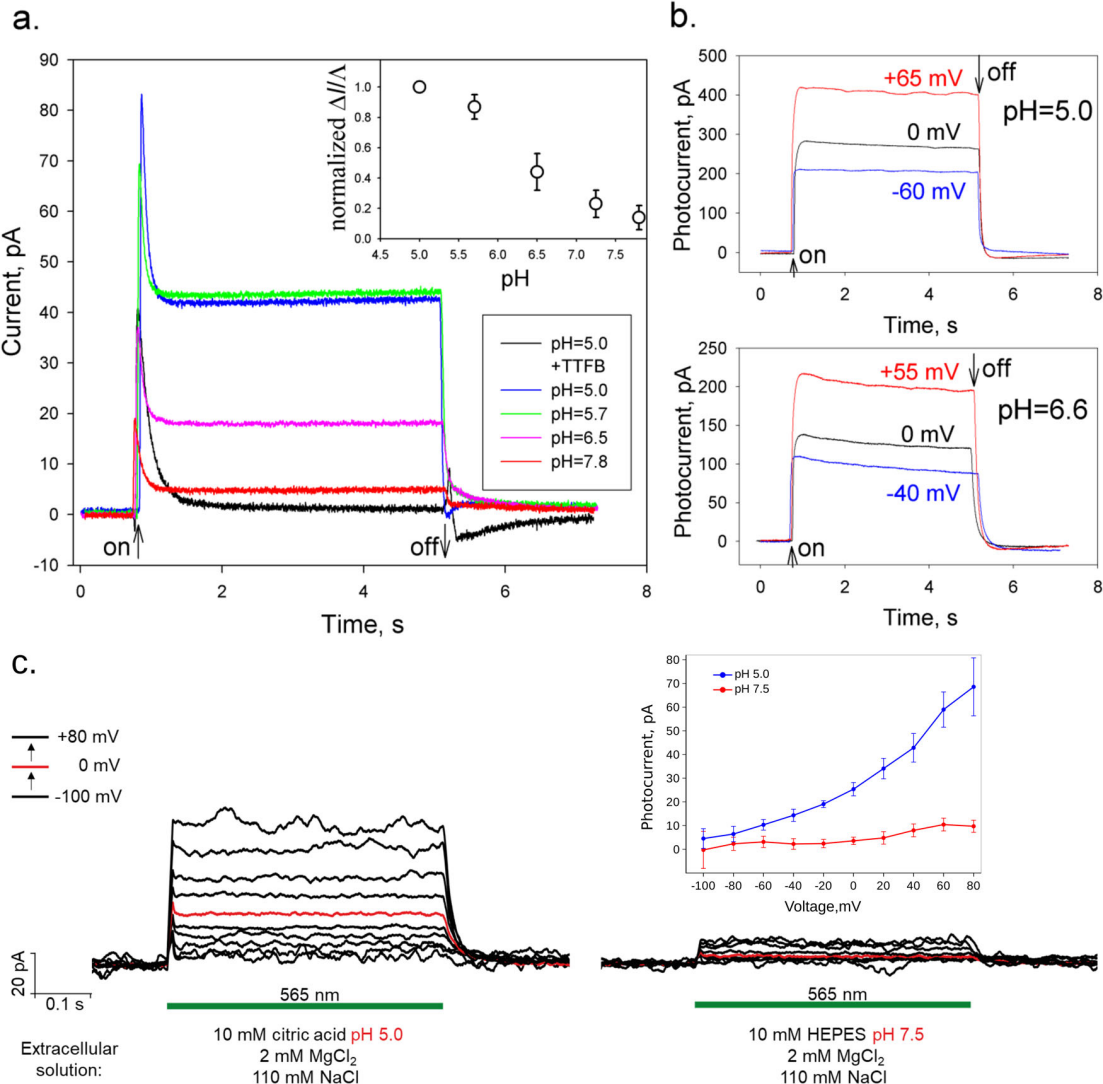
Photocurrents of proteoliposomes with SpaR adsorbed to a planar bilayer lipid membrane (BLM) at different pH and voltages. **a** The photocurrent of SpaR at different pH in the absence (black curve) and in the presence of a protonophore TTFB (other curves). The proteoliposomes adhered to one side of the BLM in a buffer containing 10 mM MES, 10 mM NaCl, pH 5.0. The photocurrents were recorded after incubation of liposomes during 1 h upon illumination of the white light without a protonophore (black line) and after an addition of 0.5 μM TTFB (blue line) at V = 0 mV (the start and the end of illumination are marked by arrows). The pH of the aqueous solution was altered by adding of different aliquots of the Tris solution. The green, purple and red lines represent the photocurrent at pH 5.7, 6.5, and 7.8, respectively. The insert shows the pH dependence of stationary photocurrent normalized on the BLM conductance. Error bars correspond to standard deviations (*n* = 4). **b** Voltage dependence of the BLM photocurrent of proteoliposomes with SpaR adsorbed to a planar BLM in the presence of 0.5 μM TTFB at different pH of the buffer solution: 10 mM MES, 10 mM Tris, 10 mM KCl, pH 5.0 or pH 6.6. The BLM was illuminated by white light during the time indicated by the arrows. The BLM conductance was 50 nS at pH 5.0 and 36 nS at pH 6.6. **c** Voltage-clamp records from one representative NG108-15 cell, expressing SpaR, with fixed intracellular conditions: 10 mМ HEPES pH 7.5, 2 mМ MgCl_2_, 10 mМ EGTA, 110 mМ NaCl. Illumination (by means of 565 nm LED) period is indicated with a green line. The extracellular solutions were varied during the patch as shown in the figure. The current-voltage dependences for one representative cell at pH 5.0 (blue) and pH 7.5 (red) of extracellular solution are shown in the insert. Error bars correspond to standard deviations during the plotted photocurrent record. The currents are normalized to a holding current. The holding potentials were from −100 mV to + 80 mV in 20 mV steps.

Next, we used a planar bilayer lipid membrane (BLM) with proteoliposomes bound to one side of the BLM^40,41^. The formation of the unilamellar lipid vesicles was verified by SAXS as described above (Supplementary Fig. 12). The curves in Fig. 3a, b shows typical traces of the BLM current upon illumination of the membranes with *Spa*R proteoliposomes at different pH values with white light. The sign of the current is the same as for BR^42^ suggesting that pumping of protons is directed inside liposomes (and correspondingly outward the cells). Without a protonophore, the current exhibited a rapid increase and a relaxation on a sub-second time scale (black curve, Fig. 3a). The addition of a protonophore 4,5,6,7-tetrachloro-2-trifluoromethyl benzimidazole (TTFB) led to the appearance of a steady-state current at pH 5.0 showing light-driven proton pumping at acidic conditions (blue curve, Fig. 3b). These conclusions are supported by the control experiment with BR-containing proteoliposomes^42^ (Supplementary Fig. 12c).

The amplitude of the *Spa*R-induced photocurrent is very sensitive to pH decreasing about ten-fold at pH 7.8 compared to pH 5.0 (red curve). The pH dependence is not the result of a variation of the TTFB activity with pH. Indeed, the amplitude of the photocurrents normalised on the BLM conductance retained the pH dependence (insert to Fig. 3a). As it is expected, similar experiments with BR-containing proteoliposomes demonstrate only a weak pH dependence of the protonophore-mediated steady-state photocurrents (20 % decrease from pH 6.0 to pH 8.0, Supplementary Fig. 14a, b). *Spa*R demonstrates a moderate dependence on the BLM voltage under acidic pH (Fig. 3b). The same is true for BR (Supplementary Fig. 14).

### Electrophysiological studies of *Spa*R

The neuroblastoma glioma (NG) 108-15 cells (88112302-1VL, Sigma-Aldrich obtained from ECACC) were transfected with the pcDNA3.1(−) vector bearing *Spa*R gene with N-terminal sequence from channelrhodopsins ChR1 and ChR2 and C terminus comprising membrane trafficking signal from potassium channel Kir2.1 and a yellow fluorescent protein variant (EYFP). The *Spa*R localization in the cells’ plasma membrane was confirmed by confocal fluorescent microscopy (Supplementary Fig. 15).

Whole-cell voltage-clamped experiments were performed. Figure 3c shows photocurrents generated by *Spa*R in the representative NG108-15 cell. We measured light-induced photocurrents in the external pH range from 4.7 to 9.0 at a number of cells expressing the SpaR gene compared to control ones. The pH of the solutions was symmetric (equal for extra and intracellular solutions). The typical photocurrent values vary from 70 to 150 pA at 100 mV applied potential at pH 5.0, 6.0 and was around 20 pA at pH 7.0, 8.0. The currents were extremely small (about several pA) at negative potentials, but their direction remained the same under all conditions. Thus, the amplitude of the photocurrents dramatically depends on pH and holding potential, but their direction did not change upon the potential of different polarity (Fig. 3c, insert). This fact confirms the proton pumping activity of *Spa*R at acidic pH, which considerably decreases at higher pH.

### Crystal structure of *Spa*R

To understand the molecular mechanisms of the *Spa*R function we crystallized the protein using the in meso approach^43–45^. The crystals were red, rod-shaped and reached ~60 μm in length. The structure of *Spa*R was solved at 2.8 Å by X-ray crystallography (PDB ID: 8ANQ, Supplementary Data 1).

Similarly to BR, SpaR forms trimers in the crystals (Supplementary Fig. 16)^45^. It is in line with the SEC and SAXS data described above. The protein protomers within the trimer are almost identical at the current resolution. Each protomer has seven transmembrane α-helices (A to G) connected with three cytoplasmic and three extracellular loops (Supplementary Fig. 17). Like in BR^46^, in *Spa*R the B-C loop forms a β-sheet, however it is shorter than that of BR. As in all known rhodopsins, the retinal co-factor is covalently bound to the K211 residue of the helix G.

The retinal binding pocket of *Spa*R is similar to that of BR (Supplementary Fig. 17). However, several residues around the β-ionone ring of the retinal are different in *Spa*R: F140, C137, Y133 and F184, compared to M145, C137, Y133 and F184 of BR, correspondingly. The presence of one additional aromatic residue F140 in the case of *Spa*R may explain the differences of the absorption spectra.

**Fig. 4:**
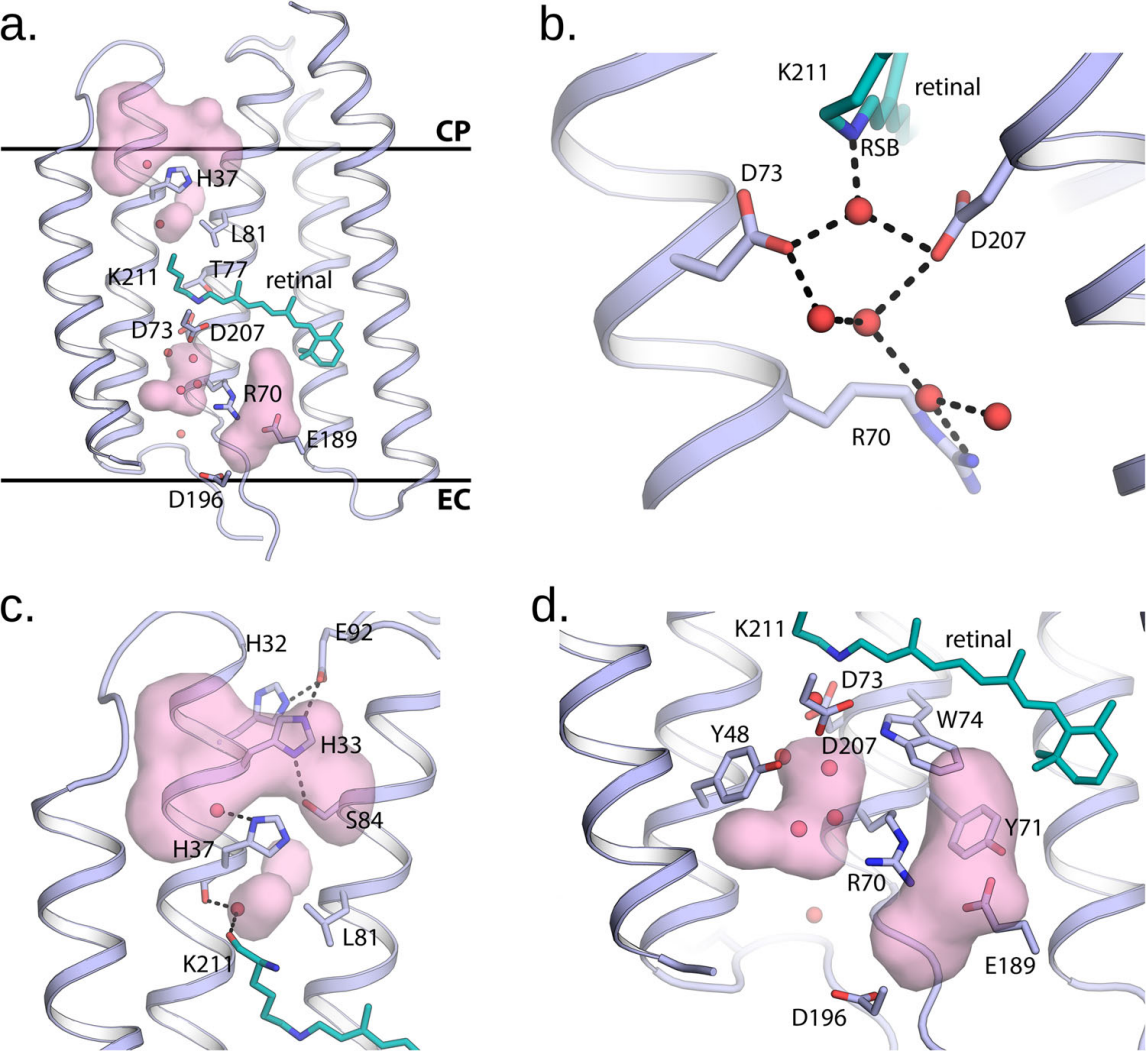
Crystal structure of SpaR. **a** Overall side view of the protein. Hydrophobic/hydrophilic membrane core boundaries are shown with grey horizontal lines. **b** The RSB region of SpaR. H-bonds in the region are shown with black dashed lines. **c** Detailed view of the cytoplasmic part of SpaR. H-bonds in the region are shown with black dashed lines. **d** Detailed view of the extracellular part of SpaR. Retinal is colored teal in all panels. The cavities are shown with pink surface.

The extracellular part of *Spa*R differs significantly from that of in BR, with two big hydrophilic cavities present in the structure, separated by a gate with R70 in the centre. The first cavity EC1 (we follow the terms introduced for ChR2^47^) protrudes from the extracellular surface of the protein down to R70 and W74 (Fig. 4d). The hydrophilic cavity is formed by E189, S121, S199, Y71, Y67 and D196. It is considerably different from that of BR (Fig. 4b), where the R82 region is separated from the bulk by a proton release group (E194-E204 pair), and then by a hydrophobic barrier from the bulk but surprisingly is similar to that of ChR2 and also resembles the hollow extracellular internal parts of other proteorhodopsins^48^ (Fig. 4c).

The second cavity EC2 is located between the RSB and R70, which is flipped to the extracellular side in opposite to BR. As in BR and many other microbial rhodopsins, D73 is stabilized by T77, while D207 is stabilized by two tyrosine residues, Y180 and Y48 (Fig. 4d).

**Fig. 5:**
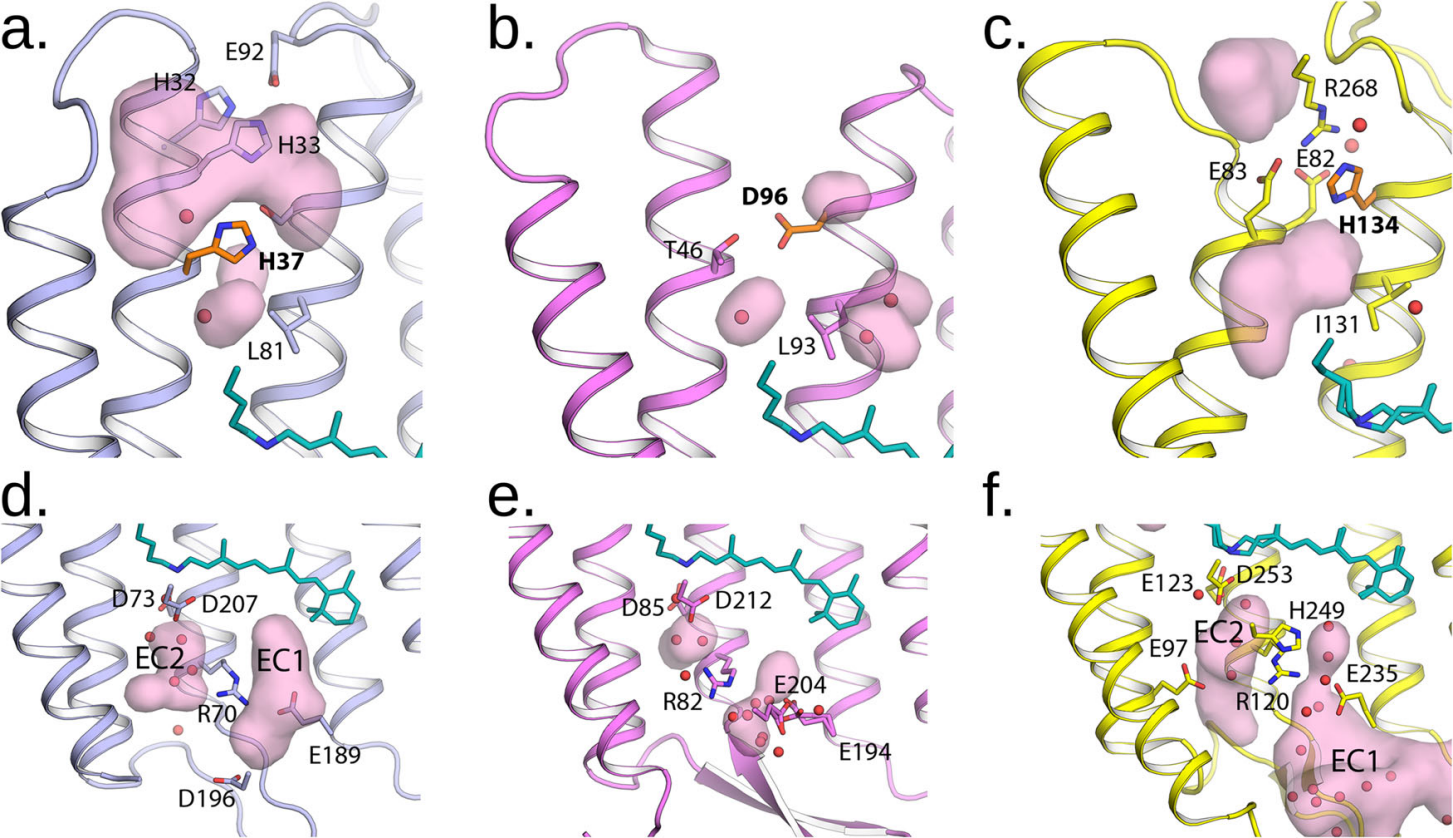
Detailed view of the cytoplasmic and extracellular parts of the rhodopsins. Detailed view of the cytoplasmic part of (**a**) *Spa*R; (**b**) BR; (**c**) ChR2. Detailed view of the extracellular part of (**d**) *Spa*R; (**e**) BR; (**f**) ChR2. Retinal cofactor is colored teal. The cavities are shown with pink surface. The central amino acid residues at the cytoplasmic side of the proteins (H37, D96, and H134) are colored orange.

Even a more unusual feature in comparison with the known rhodopsins is the organization of the cytoplasmic part of *Spa*R (Fig. 4c; Fig. 5a–c;). H37 is found at the position of T46 in BR, therefore, it is closer to the RSB than D96 in case of BR. However, it is separated from the RSB by the side chain of L81 (analogue of L93 in BR). On the other side, the H37 side chain is located within the hydrogen bond distance from S84 of the helix C. A wide cavity directly connects the cytoplasm to H37 and S84. The cavity is surrounded also by the hydrogen-bonded residues H32, H33, and E92 (Fig. 4c).

It should be noted that although the position of H37 in the cytoplasmic part of *Spa*R is similar to the position of H48 in archaeal light-driven inward proton pumps (xenorhodopsins), such as *Ns*XeR^49^ but also to the position of the key residue H134 in ChR2 (Fig. 5c). Although H37 is located at helix B, but not at helix C as the corresponding H134 in ChR2, it is side chain points at the same place. Namely, it is placed in the gate between the two cavities, similar to H134 of ChR2^47^.

Thus, compared to BR, proteorhodopsins, and other proton pumps, the *Spa*R structure is distinguished by the presence and complex organization of internal hydrophilic cavities at both cytoplasmic and extracellular sides the protein. If we compare the structure of *Spa*R with *Gt*ACR1, the anion channel, we can again note the presence of a similar structure of the extracellular gates formed by R70 (Supplementary Figs. 17, 18). Indeed, the arrangement of the pair of residues Y48 and R70 in *Spa*R is similar to the arrangement of the pair of Y72 and R94 in *Gt*ACR1 (Supplementary Fig. 18). In the cytoplasmic part of both *Spa*R and *Gt*ACR1, there is a large cavity connecting the inner part of the protein with the cytoplasm, which is not present in ChR2 (Supplementary Figs. 19, 20). In general, *Spa*R has rather ‘channel-like’ structure than that of BR and other known light-driven proton pumps.

Based on our structural, spectroscopical, electrophysiological, and site-directed mutagenesis data, the following molecular mechanism of light-driven outward proton pumping by *Spa*R can be proposed. First, since the RSB region of *Spa*R, including the H-bond pentagon formed by the RSB counterions D73 and D207 (D85 and D212 in BR, respectively) and three water molecules, are almost identical to that of BR, we suggest that the deprotonation of the RSB with the formation of the M state proceeds to D73 in the similar manner in *Spa*R and BR. Then, during the transitions through the late intermediates of the photocycle, the RSB is reprotonated from the cytoplasmic side of the protein. In the proton-pumping mode at low pH the reprotonation of the RSB with the decay of the blue-shifted M state are almost independent of the pH (in the range of 4.6–6.0). This suggests that there is an internal proton donor for the RSB. We suggest that H37 plays this role. However, we also cannot exclude that H37 is only a part of the proton donating group. At higher pH values the M decay is strongly dependent on the pH of the surrounding media, which is a sign of a direct reprotonation of the RSB from the bulk. We suggest that at pH higher than 6 the H37 residues becomes deprotonated already in the ground state of *Spa*R; therefore, it cannot play a role of proton donor and proton is uptaken from the cytoplasm through the large cavity characteristic for *Spa*R. The proton release mechanism from the RSB to the extracellular space remains elusive. We can only speculate that it proceeds differently from that in BR since the organization of the region is dramatically different in these proteins. At the same moment, the process might be similar in *Spa*R and PRs. Further investigations are needed to understand the molecular mechanism of proton transfer by *Spa*R.

### Site-directed mutagenesis of *Spa*R

To identify a pathway and key amino acids involved in the mechanism of proton transport by *Spa*R, several mutants were expressed and characterized. First, S84, homologous to the proton donor D96 of BR, was replaced by aspartic and glutamic acid. These mutants were expressed in *E. coli*, as described in Methods. Their light-induced proton pumping ability was studied in *E. coli* suspensions in salt solutions, as described in Methods. We observed a lower proton pumping of both mutants at pH 6.5 in comparison to the wild-type *Spa*R. The transient absorption changes of S84D and S84E mutants determined by time-resolved spectroscopy are shown in Supplementary Fig. 21b. The absorption maxima are 541 nm and 542 nm in S84E and S84D, respectively (Supplementary Fig. 21a). As expected, introducing a carboxylic residue into the helix C provided a faster M-decay rate, and as a result, about two times faster photocycle at pH 8.0 in both S84D and S84E mutants was observed. Nevertheless, the photocycle remained rather slow (about 1 s). This fact confirms that *Spa*R uses a not yet described mechanism of proton transport.

In addition, we designed seven additional mutations where H37 was substituted by A, K, R, L, F, Y and N, one double mutant where H32 and H33 were both replaced by A, and a D73N mutant analogous to D85N mutant of BR. We measured the light-induced pH changes of suspensions of *E. coli* expressing these mutants at pH 6.5 in the NaCl unbuffered solution and with the addition of CCCP (Supplementary Fig. 22). The D73N mutant was phenotypically similar to the D85N mutant of BR with blue color (instead of red - normal for SpaR) and showed almost no proton pumping activity at pH 6.5. This fact confirmed that in *Spa*R D73 acts as the primary proton acceptor from the RSB^50^.

### Zinc inhibits outward proton pumping of *Spa*R

**Fig. 6:**
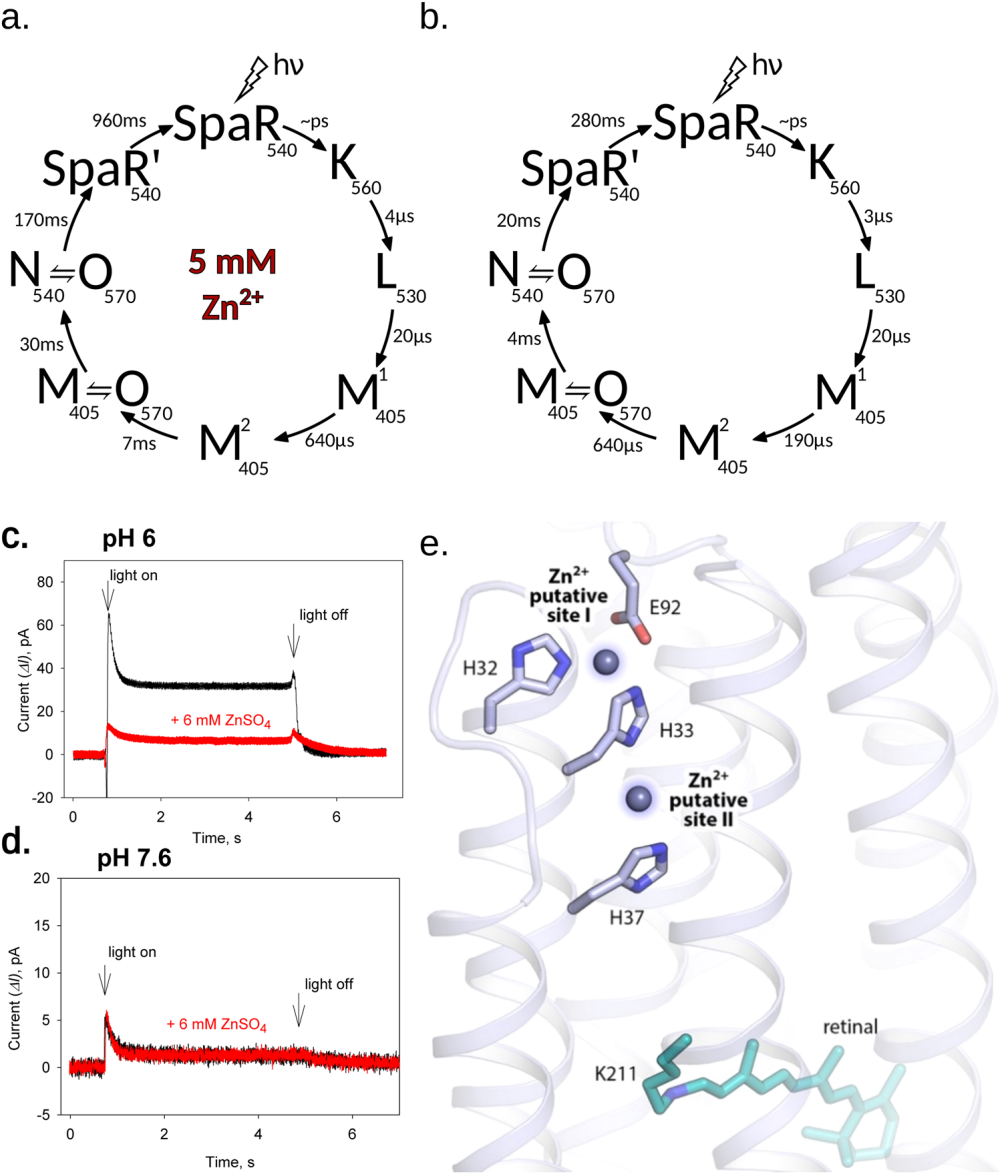
Zn^2+^ influence on SpaR function. Photocycles of solubilized (**a**) SpaR at pH 5.0 and zinc concentration 5 mM; (**b**) SpaR at pH 5.0 without zinc. **c**, **d** Effect of zinc ions on the photocurrents of proteoliposomes with SpaR adsorbed to a planar bilayer lipid membrane (BLM) at pH 6.0 (**c**) and pH 7.6 (**d**) in the presence of 0.5 uM TTFB protonophore. **e** The putative zinc binding sites in SpaR. The cytoplasmic side of SpaR is shown. Two putative zinc binding sites are proposed. The putative zinc atoms are shown by blue spheres. Retinal cofactor is coloured teal.

Since several *Spa*R-like rhodopsins were found in zinc-dependent bacteria^4,14,51–55^ we studied the influence of Zn^2+^ on the *Spa*R function. This influence could not be referred to the unspecific binding of Zn^2+^ to the *Spa*R or to the binding of Zn^2+^ to the His-tag of the recombinantly-expressed *Spa*R (as was studied in^56^) due to the low pH (lower than pK_His-tag_) and the dramatic dose-dependent influence of Zn^2+^ on *Spa*R photocycle (Fig. 6, Supplementary Figs. 23, 24). The comparison of the photocycles with and without Zn^2+^ measured at pH 5.5 is shown in Fig. 6a, b. Two latest intermediate states of *Spa*R are highly sensitive to the presence of zinc at the concentrations higher than 3 mM (Supplementary Fig. 23). Zn^2+^ slows down the final stages of the photocycle. The changes of the absorption spectrum of RSB during titration at low pH and pK_D73_ in the presence of 5 mM Zn^2+^ is almost the same as without Zn^2+^ (Supplementary Fig. 7). The dissociation constant Kd_Zn2+_ in millimolar range was estimated from the dose-dependent showering of duration of the photocycle (Supplementary Fig. 24). The *Spa*R photocycle is not sensible to Cd^2+^ and Cu^2+^ ions. The Fe^2+^ slightly changes the duration of photocycle, but in comparison to Zn^2+^ the influence is negligible (Supplementary Fig. 25). An increase of the melting temperature of the protein in the presence of Zn^2+^ by 10 °C at pH5.5 and by 20 °C at pH7.0 (Supplementary Fig. 26) could be referred to the stabilizing of the protein structure in the presence of Zn^2+^ and can give more evidence of specific Zn^2+^ interaction with *Spa*R.

We also performed the studies of the photocurrents of proteoliposomes with *Spa*R adsorbed to a planar bilayer lipid membrane (BLM) at different pH in the presence of 6 mM of Zn^2+^. We compared the results obtained without and in the presence of Zn^2+^ at different pH (Fig. 6c, d). Zn^2+^ inhibited proton pumping at pH 6.0, photocurrent decreased by 4.25+/−0.95 times (*n* = 4). This is the first observed dramatic influence of Zn^2+^ ions on the rhodopsin function. Importantly Ca^2+^ or Mg^2+^ cations were of no inhibitory effect showing high selectivity of the effect (Supplementary Fig. 27).

The combined results of the spectroscopy studies and functional tests allow us to speculate that Zn^2+^ binding blocks the reprotonation of the RSB associated with the late states formation and thus inhibiting the proton-pumping activity of *Spa*R. We suggest that Zn^2+^ binds at the cytoplasmic part of the proton translocation pathway, since this region is associated with the RSB reprotonation. An important question arises what is the mechanism of this binding. Figure 6e shows two putative binding sites in the structure of *Spa*R based on the presented structure of the protein. The first site might be formed by H32, H33, and E92. The second possibility is for Zn^2+^ to bind between H33 and H37. In both cases Zn^2+^ can affect the protonation state of H37, which, in its turn, might result in blocking the proton-conductive pathway or the switch. Importantly, both proposed putative Zn^2+^ binding sites are freely accessible from the cytoplasm.

### *Spa*R as potential optogenetic tool

The protein might be potentially used as an optogenetic tool. The optogenetic application of archaeal proton outward pumps has been demonstrated^57^. Their pumping is stable in a wide pH range^3, 57^. In opposite, proteorhodopsins are pH sensitive. Moreover, proteorhodopsins and mirror proteorhodopsins are complementary pH selective proton pumps. Therefore, we can expect that proteorhodopsins can be used as optogenetic tools for the studies of those biological functions/dysfunctions associated with different pH states of the cells and organelles. For example, *Spa*R expressed in lysosomes may change their acidity only when they are acid but has no influence on them when the lysosomal pH is neutral or alkaline. In opposite, a normal proteorhodopsin will affect the pH of lysosomes only when they are neutral or alkaline. Therefore, for instance, the pair of a proteorhodopsin and a mirror proteorhodopsin co-expressed in a lysosome may not only probe their state but also control their pH dependent functions selectively. To show the feasibility of this approach, we expressed *Spa*R in lysosomes of HEK293T cells. We demonstrated a selective expression of the protein in lysosomes and showed that *Spa*R can modify their pH (Supplementary Fig. 28), as it is done with archaeal Arch3^58^. Thus, it is important to experimentally verify the potential of mirror proteorhodopsins as a pH selective optogenetic tool.

## Discussion

It would be unwise to consider that our data are sufficient to specify the biological roles of mirror proteorhodopsins (mPRs) and, in particular, their role in pathogens. Nevertheless, we would like to discuss several points, which could help generate a working hypothesis of their roles.

First, our analysis shows that the clade of *Spa*R-like rhodopsins comprises only Gramm-negative bacteria and those belonging to genuses *Sphingomonas, Patonea* and *Pseudomonas* (Supplementary Fig. 4). Many species of *Patonea* and *Pseudomonas* are well known as pathogens, first of all involved in hospital-acquired infections.

Second, in many cases where the host of a mirror proteorhodopsin is known, our analyses of literature data revealed that the host bacteria are pathogenic (Supplementary Fig. 3). In particular, *Spa*R*, Pa*R and *Psp*R are from *Sphingomonas paucimobilis, Pantoea ananatis* and *Pseudomonas putida*, respectively. The hosts are well-known as multidrug-resistant hospital acquired pathogens or potential pathogens^12,59–66^. Some species of *Sphingomonas*, especially *S. paucimobilis*, are found in hospital equipment and various types of clinical specimens. Already in 1991, at least 18 cases of infections (six cases of bacteremia, two leg ulcers, four peritonitis, brain abscess, cervical adenopathy, splenic abscess, respiratory, urinary infections, and meningitis) caused by this bacterium were reported^67^. Interestingly, one of the features of *Sphingomonas* is their biodegradative and biosynthetic capabilities. *Sphingomonas* are used for a wide range of biotechnological applications, from bioremediation of environmental contaminants to the production of extracellular polymers such as those used in the food and other industries^68–70^.

*Pantoea* is also known to form associations with humans and also a variety of hosts, including plants and insects^10,71–73^. *Pantonea* is also used in industry for bioremediation, herbicides degradation and other toxic products^74–77^. The microbe possesses plant growth-promoting capabilities and is used in agricultural applications^10,78^. Although often thought of as a plant pathogen^60^, *Pantoea* is frequently found in hospital environments^72,79–81^. For instance, *Pantanea ananatis* has been known for a long time as a cause of opportunistic infections in humans^66,71^.

*Pseudomonas putida* was also isolated from patients who have acquired infections in hospital environments^82–84^. Infections caused by *P. putida* are reported in immuno-compromised patients, including cancer patients^65,85–87^. A number of *P. putida* strains colonize rhizosphere of plants and promote the growth of plants^88–91^. *P. putida* strains are highly efficient in metabolizing a wide range of biogenic and xenobiotic compounds^92,93^. The remarkable biochemical versatility of *P. putida* is used for its application in industrial biocatalysis^87,94–98^.

Third, *Spa*R-like rhodopsins *Spa*R*, Pa*R, and *Psp*R are pumping protons only at acidic pH. We showed this directly in the case of *Spa*R. Currently available data allows concluding that *Pa*R and *Psp*R of two other groups of the clade (which have a DTG motif) also pump protons. Unfortunately, in the case of these two proteins, pH dependence of pumping was not directly studied. The measurements of the pumping activity were done with the proteins expressed in *E. coli* (and also with spheroplasts in the case of *Psp*R) placed in unbuffered solution^3^. Nevertheless, taking into account the high sequence and key amino acid similarities (Supplementary Fig. 2), and, importantly, the similarities of pH dependences of the photocycles of *Pa*R, *Psp*R^4^, and *Spa*R we conclude that also *Pa*R and *Psp*R rhodopsins pump protons only at acidic pH. Actually, the duration of the photocycles of both proteins, similar to that of *Spa*R (Fig. 2a, c), dramatically increases at pH > 6÷6.5^4^.

Fourth, considering such pH dependence we speculate that some bacteria may use mirror proteorhodopsins to stabilize pH inside the cells. It is known that inflamed and infected tissues and also other habitat environment are often acidic^99^. Indeed, acidification is a hallmark of inflammatory processes^100–103^. The corresponding increase of proton concentrations in the extracellular space (where pH values as low as 5.5 are observed) is often associated with the inflammatory immune responses to bacteria in peripheral tissues^104^. Therefore, it cannot be excluded that outward proton pumping by *Spa*R-like mirror proteorhodopsins may help to balance cytoplasmic pH when bacteria enter the host tissues.

Such pH dependence could occur due to the pK of His37 (the pK value of the histidine side chain without surrounded interactions is 6,0). The proton-conductive pathway involves H^+^ interaction with the His37 probably acting as a gate at the entrance to the cavity. If the large hydrophobic amino acid residues Phe and Leu are put instead of His37 they block the SpaR H^+^ pumping activity (Supplementary Fig. 22) obviously because of the lack of sites for H^+^ to interact with. In contrast, the small hydrophobic Ala residue on the place of His37 doesn’t block the pumping activity completely probably due to the enough space for H^+^ and water molecules at the gate on the H^+^ pathway. Tyr possessing the OH-group (pK = 10,0) could interact with the H^+^ and/or water and also doesn’t blocks the pathway completely. Polar aminoacid residues Arg (pK = 12,5), Lys (pK = 10,8) and Asn could mimic the His37 in sense of interaction with water and H^+^ and if present instead of His37 don’t dramatically influence the H^+^ pumping activity. Double mutant of H32A, H33A shows only slight lowering of the H^+^ pumping activity – these His residues are in the cavity and probably not acting as the His37 gate. But slight influence shows that H^+^ is interacting with the His32 and the His33.

And what about zinc? Is zinc dependence of mirror proteorhodopsins related to the behaviour of the hosts and their virulence? Zinc is a major element necessary for the function of all cells and is the second most abundant transition metal in humans. Apparently, it plays crucial roles in many facets of the immune system^18,21–23^. Zinc is also essential for the growth of pathogenic microorganisms and is involved in the regulation of various virulence factors. Additionally, zinc is necessary for infection and colonization of pathogenic microorganisms in the host. Biofilm formation is important for the survival of bacteria in hostile environments, since bacteria within a biofilm are usually more resistant to antibiotics and disinfectants. Zn^2+^ has been shown to play a role in the ability of bacteria to produce a biofilm. It was found that Zn^2+^ depletion via metal chelation specifically prevented biofilm formation of some bacteria^105^. As far as zinc deficiency is characteristic for aged population^24^ it could be important for the age-dependent increase of hospital infections particularly caused by the *Spa*R-like rhodopsin’s hosts^20,106,107^.

Next, zinc is an imperative micronutrient required for optimum plant growth. Plants can uptake zinc as divalent cation but only a tiny portion of total zinc is present in soil solution in a soluble form^53,54,108–111^. The rest of zinc is in the form of insoluble complexes and minerals^91,112^.

Many *Sphingomonas, Pantoea*, and *Pseudomonas* are known as zinc-solubilizing and zinc-resistant^14,113–115^. It has been shown, that also *Pantoea dispersa* comprising a gene of rhodopsin similar to *Pa*R, and *Pantonea ananatis* with *Pa*R are solubilizing zinc, which promotes plant growth^10,51^. Most heavy metals, including zinc, are immobilized in soil. Some types of plant growth-promoting rhizobacteria (PGPR) increase the heavy metal uptake into plants by solubilizing them^90,97,108,114^. Particularly, *Pseudomonas putida* is solubilizing zinc in the sediment and is zinc resistant^113,116^. It was shown that opportunistic *Pantoea ananatis* resistant to antibiotics can solubilize zinc^10^.

Zn^2+^ is believed to be one of the most potent inhibitors of proton channels. The inhibitory action of Zn^2+^ is derived from metal ion binding to histidine residues, and also to the thiol group of the cysteine residues^117^. Interestingly, as we showed, there are at least two putative histidine-rich Zn^2+^ binding sites in *Spa*R (Fig. 6e.). Notably, the proton translocation inhibitory role of zinc is well-known in biology. In the respiratory chain, Zn^2+^ acts on the main proton-driving force generators by inhibiting reduction and protonation of quinone and/or the translocation of protons in complexes I and III in mitochondria. Also, Zn^2+^ inhibits NADH dehydrogenase in *E. coli* and inhibits the activity of mitochondrial cytochrome C oxidase (COX) as well as different bacterial COXs^118^. There are several different mechanisms by which Zn^2+^ binding could slow proton transfer steps. The binding of Zn^2+^ ions to the H, E, or D residues could directly and/or indirectly affect proton transfer pathways, e.g., via binding to the residues which are directly involved in the proton release/uptake or to the residues which can electrostatically increase the potential energy for the proton transfer through the pathway. The binding of the residues from different helices could, in principle, hinder the conformational changes coupled to the proton release. Beside the electrostatic effects on the residues, Zn^2+^ can directly restrict water chains which provide the pathway for the proton^118^. Besides, zinc ions usually compete with protons for histidine residues, as a result of which the inhibitory effects of zinc are more noticeable at alkaline pH than at acidic ones.

The acidic pH is necessary for divalent metal ions, particularly zinc, solubilization by bacteria^52,54^, and in soils with low pH the solubility of zinc increases^55^. So we suggest that mirror proteorhodopsins in plant growth-promoting bacteria (PGPB) may participate in the promotion of zinc mediated plant growth. Indeed, mPRs are pumping protons to extracellular environment and additional acidification may facilitate chemical reactions (like, for instance, ZnO + 2H^+^ = Zn^2+^ + H_2_O) of zinc solubilization to promote plant growth. Disclosing of the biological role of mirror proteorhodopsins requires further in vitro and in vivo comprehensive studies of a representatives of the clade and their role in the host and their interactions with the environment and humans. The studies are highly motivated also by the fact that the corresponding bacteria are of high interest to microbiology, biotechnology, medicine and maybe in optogenetics.

## Methods

### Phylogenetic analysis and sequence alignment

For the phylogenetic tree of microbial rhodopsin proteins, 33 rhodopsin sequences were aligned using MUSCLE. Phylogenetic reconstruction was conducted using Jalview 2^119^ (average distance, BLOSUM62 score matrix).

### Cloning

The *Spa*R coding DNA sequence (UniProt ID: A0A0C9NB29) was optimized for *E. coli* Class II expression using the *E. coli* Codon Usage Analyzer 2.1^120^. 5’ RNA termini including plasmid vector’s UTR (untranslated region), was also optimized to reduce the probability of hairpins formation and to minimize the free energy with the use of RNA WebServer (Institute for Theoretical Chemistry, University of Vienna^121^). The gene of *Spa*R was assembled by two stage PCR with the use of 14 overlapping short oligonucleotides^122^ (Evrogen, Russia) developed by DNAWorks v3.2.4 software^123^. The constructed gene sites was introduced into the pET32b expression vector (Novagen) via *Nde*I and *Xho*I restriction sites so the gene obtained 3´ extension coding polyhistidine tag. The mutant variants were prepared by site-directed mutagenesis and verified by sequencing (service provided by The Institute of Bioengineering of Federal Research Center “Fundamentals of Biotechnology” of Russian Academy of Sciences (Moscow, Russia)).

### Heterologous expression, solubilization, and purification

*E. coli* cells of strain C41 (DE3) (Lucigen, USA) were transformed with the constructed plasmid vector. The transformed cells were grown at 37 °C in shaking baffled flasks in an autoinducing medium ZYM-5052^124^, containing ampicillin (200 mg/l), 10 μM all-trans retinal (50 mM stock solution in ethanol) and 1 mM isopropyl-b-D-thiogalactopyranoside (IPTG) was added in 1–3 h before harvesting. Than the cells were collected by centrifugation at 13000 g for 10 min. The collected cells were collected by centrifugation at 4500 g for 10 min and disrupted in M-110P Lab Homogenizer (Microfluidics, USA) at 25,000 psi in a buffer containing 20 mM TRIS-HCl (pH 8.0), 150 mM sodium chloride, 0.25% Triton X-100, 0.1 mM PMSF (phenylmethane sulfonyl fluoride, Amresco, USA). The membrane fraction of the cell lysate was isolated by ultracentrifugation at 100,000 g for 1 h at 4 °C. The pellet was resuspended in a buffer containing 50 mM TRIS(HCl)pH 8.0, 500 mM NaCl, 1% DDM (Anatrace, Affymetrix, USA) and stirred overnight at 4 °C for solubilization. The insoluble fraction was removed by ultracentrifugation at 100,000 g for 1 h at 4 °C. The supernatant was loaded on a Ni-NTA column (Qiagen, Germany), and the protein was eluted in a buffer containing 50 mM NaH_2_PO_4_/Na_2_HPO_4_ (pH 7.5), 100 mM NaCl, 300 mM imidazole, and 0.1% DDM. The eluate was concentrated by means of Stirred Cell (Amicon) with 30 kDa MWCO membrane. Then, the protein was additionally purified by size-exclusive chromatography using Superdex 200 Increase 10/300 GL column (GE Healthcare Life Sciences, USA). Finally, the fractions of the protein with a maximal peak ratio A_280_/A_540_ were concentrated to 40 mg/ml for crystallization.

### Small-angle X-ray scattering (SAXS) measurements with solubilized

*SpaR* were performed in Grenoble (France), on the beamline BM-29 (synchrotron ESRF)^125^. IFT-fits and Patterson functions P(r) were performed using the Gnom program from ATSAS software suite^126^. An accurate SAXS data treatment for solubilized membrane proteins requires explicit accounting of the detergent belt contribution to the scattering profile^127,128^. For this purpose, the MEMPROT program^33^ was used to model the detergent corona around a protein and to fit the experimental SAXS data using this model. The sample of *Spa*R after gel-filtration was concentrated to ~5 mg/ml, and then, dialysis was performed for 12 h in a buffered solution 100 mM NaCl, 0.5 mM EDTA, 0.01% NaN_3_, 20 mM TRIS(HCl) pH7.2, 0.05% DDM. The SAXS profile is presented in Supplementary Fig. 6. For the trimer model, the optimal parameters of the detergent corona found by the fit are: a = 34.5 Å, b = 5.0 Å, t = 6.5 Å, ε = 1.19, φ = 79° (we used Adaptive shape algorithm type 2 (MBJP) and Crysol 3), that corresponds to the 400 detergent molecules around the SpaR trimer.

Single lipid vesicles preparation for pump activity measurements were as described previously in ref. ^129–131^. Phospholipids (asolectin from soybean, Sigma-Aldrich) were dissolved in CHCl_3_ (chloroform ultrapure, PanReac AppliChem) in a glass flask. The flask was then connected to a rotor evaporator until the total evaporation of the solvent under vacuum and the formation of a thin lipid film on the sides of the flask. The residual solvent was removed using a vacuum pump overnight. The dried lipids were resuspended at a final concentration of 1% (w/v) in 0.1 M NaCl supplemented with 2% (w/v) sodium cholate. The mixture was clarified by sonication at 4 °C, and *Spa*R was added at a protein/lipid ratio of 7:100 (w/w). The detergent was removed by overnight stirring with detergent-absorbing beads (Amberlite XAD-2, Supelco). The mixture was dialyzed against 0.1 M NaCl (adjusted to a desired pH) at 4 °C for 1 day (four 200 ml changes) to obtain a certain pH, or against 50 mM NaH_2_PO_4_/Na_2_HPO_4_ (pH 7.5), 0.1 NaCl (for BLM and SAXS studies).

Small-angle X-ray scattering (SAXS) measurements with SpaR proteoliposomes were performed on SAXS instrument Rigaku MicroMax-007HF at MIPT (which previously was used and was described in works^132–134^) to verify the formation of ULV. A standard model of the bilayer with a symmetric step EDP function was used for description of the SAXS scattering profile for liposomes. A SasView (version 4.2.1) program was used for modelling. In this model the inner layer corresponds hydrophobic tails and outer layers correspond to hydrophilic ‘heads’ of lipids. The electron density contrast *Δρ(z)* = *ρ(z) - ρ*_*buf*_ was described in an arbitrary scale and the density contrast of the hydrophilic layers was taken 1 (in other words, we estimated *Δρ(z) / Δρ*_*MAX*_ by fit of SAXS data, see Supplementary Fig. 12d and Supplementary Table 2). According to ref. ^134^, phosphatidylcholine (PC) is the main PLs in soybean lecithin (>55%). According to ref. ^135^, for common PCs such as DPPC, DMPC, DOPC and EPC, the thickness of a hydrophilic layer equals 9 Å. Interestingly, in the case of DPPC at 20 ˚C and 50 ˚C (i.e., a liquid crystal and a gel-phase, respectively) this value was the same. Given the aforementioned information, we fixed the thickness of the hydrophilic layers at 9 Å. We used the Shultz distribution to describe size polydispersity.

### Measurements of pump activity in E. coli suspensions and lipid vesicles

The single lipid vesicles were prepared as described above. The *E. coli* cells expressing *Spa*R were collected by centrifugation at 4500 g for 10 min and washed three times with an unbuffered salt solution (100 mM NaCl or 100 mM KCl), with 30 min intervals between the washes to allow exchange of the ions inside the cells with the bulk. After that, the cells were resuspended in 100 mM NaCl solution (or 100 mM KCl solution) and adjusted to an OD600 of 8.5.

A pH meter (S20 SevenEasy™, Mettler Toledo) was used to record the changes in the pH of the suspension of *E. coli* cells or lipid vesicles in response to illumination. The sample (3 mL) was placed in a glass flask with a Teflon magnetic stirrer inside, kept at 0 °C. The cells and the lipid vesicles were illuminated for 5 and 10 min, respectively, using a cold-light reflector lamp (Olympus KL2500-LCD, 250 W). The measurements were repeated under the same conditions after the addition of 30 µM protonophore CCCP (carbonyl cyanide m-chlorophenyl hydrazone).

### Spectroscopic characterization and time-resolved absorption spectroscopy

The laser flash photolysis setup was similar to that described by Chizhov et al.^35^. The excitation/detection systems consisted of Nd:YAG laser (Quantel, France) generating pulses with an energy of ~2 mJ and a duration of 4 ns at a wavelength of 500 nm, LSH-150 monochromators (LOT, Germany), Xe-arc lamp light source (75 W, Hamamatsu, Japan), photomultiplier tube (PMT) detector (R12829, Hamamatzu), and two digital oscilloscopes (Keysight DSO-X 4022 A). The samples (5 × 5 mm spectroscopic quartz cuvette; Hellma GmbH & Co.) were placed in a thermostated house between two collimated and mechanically coupled monochromators (1/8 m model 77250, Oriel Corp.). The probing light (Xe-arc lamp) passed the first monochromator sample and arrived after a second monochromator at a PMT detector. The current-to-voltage converter of the PMT determines the time resolution of the measurement system of ca. 50 ns (measured as an apparent pulse width of the 5 ns laser pulse). Two digital oscilloscopes were used to record the traces of transient transmission changes in two overlapping time windows. The maximal digitizing rate was 10 ns per data point. The transient absorption changes were recorded from 10 ns after the laser pulses until full completion of phototransformation. At each wavelength, 25 laser pulses were averaged to improve the signal-to-noise ratio. The quasilogarithmic data compression reduced the initial number of the data points per trace (~50,000) to ~600 points evenly distributed in a log time scale, giving ~100 points per time decade. The recording of the absorption changes was started 700 ns after the laser pulse before the end of the photocycle, at wavelengths from 330 to 730 nm with a step of 10 nm. The absorption spectra of the samples were measured before and after each experiment on a UV-2401PC spectrophotometer (Shimadzu, Japan). The obtained data were analysed by global fitting using the MEXFIT software^35^.

### SpaR expression in NG108-15 cells

The eucariotic codon–optimized SpaR gene was also assembled by PCR with the use of 14 overlapping short synthetic oligonucleotides (Evrogen, Russia). The gene was cloned into the pcDNA3.1(−) vector bearing an additional membrane trafficking signal from Kir2.1 and an EYFP variant. The gene was cloned under the T7 promoter via *Kpn*I and *Not*I restriction sites. The sequence was verified by sequencing. The NG108-15 cells were grown under 5% CO_2_ at 37 °C in Dulbecco’s Modified Eagle’s Medium (DMEM) (Thermo Fisher Scientific, USA), containing 10% heat-inactivated fetum bovine serum, 1% penicillin/streptomiccin (Thermo Fisher Scientific, USA), 2 mM GlutaMax (Thermo Fisher Scientific, USA), and 10 mM HEPES pH 7.4 (Thermo Fisher Scientific, USA). Cells were grown in 24-well plate (Corning Incorporated, USA) till confluency ~90%. Then they were transfected by Lipofectamine™ LTX Reagent supplied with PLUS™ Reagent (Invitrogen, USA) in accordance to the manufacturer’s protocol (the amount of Lipofectamine™ LTX Reagent per well increased to 1,5 µL). After 4 h of incubation under 5% CO_2_ at 37 °C, the transfection medium (Opti-MEM Reduced Serum Media, Thermo Fisher Scientific, USA) was changed back to the growth medium. After 12–24 h the cells were seeded on the glasses for further patch-clamp and microscopy experiments, which were conducted after 3–12 h.

Whole-cell patch-clamp recordings on NG108-15 cells expressing *Spa*R were performed with Scientifica LASU, Axon Digidata 1550 A, Multiclamp 700B. Patch pipettes with resistances of 3 to 6 megohms were fabricated from thin-walled borosilicate glass (GB150F-8P) on a horizontal puller (Model P-1000, Sutter Instruments, USA). Photocurrents were measured in response to light pulses using light diode with a wavelength of 565 ± 20 nm (pE-100 565 nm, CoolLED. UK). Light-induced photocurrents, their potential dependences as well as photocurrent dependence on pH were similar for a number of cells (>10) which were expressing SpaR. Control cells (>7) which were not subjected to transfection by the plasmid bearing the SpaR gene showed no light-induced photocurrents.

### Planar bilayer lipid membrane (BLM) experiments

The BLM was formed from a solution of 1,2-diphytanoyl-sn-glycero-3-phosphocholine and 1,2-dimyristoyl-sn-glycero-3-ethylphosphocholine in n-decane (20 and 0.4 mg/ml) on a 0.6 mm aperture in a Teflon septum separating the experimental chamber into two compartments of equal size (volumes, 3 ml). The electrical current was measured with two AgCl electrodes placed into the solutions on the two sides of the BLM via agar bridges, using a Keithley 428 amplifier (Cleveland, Ohio, USA). A protonophore TTFB (tetrachlorotrifluorobenzimidazole) was a gift of Lev Yaguzhinsky (Moscow State University). BLMs were exposed to continuous illumination with a halogen lamp (“Novaflex”, World Precision Instruments, USA) providing an incident power density of 0,8 W/cm^2^. The photocurrents were recorded after the incubation of liposomes during 1 h upon illumination of the white light. pH of the aqueous solution was altered by addition of different aliquots of the Tris solution.

### Electrometric time-resolved measurements of the membrane potential

The kinetics of the transmembrane potential difference ΔΨ in lipid vesicles with embedded microbial rhodopsin in response to light was studied using direct electrometric measurements with high temporal resolution (100 ns), described in detail in ref. ^129,136–140^. The measured potential difference is linearly related to the value of the membrane potential generated by the light-sensitive protein in response to illumination. The experimental setup was a Teflon cell with two symmetrical cylindrical compartments, between which there was a hole of 4 mm in diameter. The hole was covered with a colloidal (nitrocellulose) film soaked in a solution of azolectin in n-decane. The membrane must be thin enough so that rapid charge movements can be recorded. Both sections of the cuvette were filled with a 25 mM HEPES (pH 7.5) or MES (pH 5.5). The measurements were made using two silver chloride electrodes on either side of the colloidal film and a high time resolution voltmeter. For this, the adhesion of lipid vesicles with the protein under study to the surface of the colloidal phospholipid film (membrane) was ensured in the presence of magnesium ions and with stirring. An Nd-YAG laser (YG-481, λ = 532 nm, pulse half-width 12 ns, energy up to 40 mJ; Quantel) was used as a source of pulses (the beam was fed through a window in the cell and was focused on the membrane). When illuminated, rhodopsin creates a potential difference ΔΨ on the vesicle membrane, which is proportionally distributed also to the measuring membrane, and therefore, it can be registered with time resolution. As a rule, the measuring membrane has a high resistance of 2–3 GΩ, and the laser flash induced membrane potential difference ΔΨ decreases in dark with a time constant of the order of several seconds due to the passive membrane discharge. This passive proton leakage through the membrane has the characteristic time τ ~ 500–1000 ms which is much more longer than the protein photocycle duration. This passive leakage makes the next iterations of the measurements possible. The measurements performed with the use of the same preparation of lipid vesicles with incorporated SpaR (analogously experiments with ESR^141^ and heliorhodopsin^142^) were reproducible and showed the analogous character and amplitudes.

### Confocal fluorescent microscopy

To obtain the images of NG108-15 cells expressing *Spa*R-EYFP in the plasma membrane, an inverted scanning confocal fluorescence microscope LSM780 (Zeiss, Jena, Germany) was used. The glass with the transfected cells was placed in an imaging dish with a transparent bottom (35 mm in diameter). The experiments were carried out using a 100x oil immersion objective (numerical aperture (NA) 1.46) and autofocus; the image size was 1024 × 1024 px (141 × 141 µm). Excitation of the *Spa*R-EYFP was carried out by 488 nm argone laser (Lasos, Jena, Germany). The resulting images were processed using the ZEN software (Zeiss, Germany) and the ImageJ, open-source image analysis and processing software (version 1.52 u).

### Optogenetic experiments

Imaging was done using an inverted confocal LSM780 microscope (Carl Zeiss, Germany), 63x (NA = 1.4, oil immersion) objective. An optical fiber (400 µm in diameter) guided through the microinjection needle holder was placed just above the cells of interest by means of microinjection micromanipulator InjectMan NI2 (Eppendorf). LED590 (ThorLabs M590F2, 590 nm emission maximum) was used with a power of 16 mW/mm^2^ (ThorLabs Thermal Power Sensor Head S302C measured) in all experiments, excluding those with a LED power variation (ThorLabs 4-Channel LED Driver, DC4104). During the experiments human cells HEK293T (ECACC 12022001) were placed in Tokai Hit CO_2_-incubator (model INUBG2H-ELY), HEPES (pH 7.4) for the final concentration 25 mM was added, 50 nM Bafilomycin A1 (B1793, Sigma-Aldrich) was added 12 h before optogenetic lysosome acidification. For time series with light illumination *λ*-mode was used. Only pHluorin fluorescence intensity was excited by a 488 nm laser. The emission was measured in a CLSM *λ*-mode using a 34-channel QUASAR detector (Carl Zeiss, Germany) set to a 488–545 nm range to avoid LED590 detection. Time curves were obtained using ZEN software by Zeiss after performing spectral linear unmixing processing. The time curves were averaged for different cells in the field of view. LED590 emission, which added intensity to the GFP spectrum, was obtained from the background ROI and then subtracted manually from time curves of lysosomes ROI to produce pure pHluorin fluorescence intensity time curves.

### Estimation of protein denaturation temperature

Protein melting temperatures were determined as maxima of the first derivatives of the 330 nm *Spa*R intrinsic fluorescence during slow heating at the rate of 0.5 °C per minute by the nanoDSF Prometheus Panta instrument (Nanotemper, Germany). Purified (>98%) protein at concentration of 0.5 mg/ml was used in 20 mM Tris(HCl) pH5.5 or pH7.0, 150 mM NaCl, 0.05% DMM, 0 or 10 mM ZnCl_2_ (Sigma-Aldrich, #229997). The data analysis and graphs plotting were performed by means of Panta Analysis software (Nanotemper, Germany).

### Crystallization

The crystals of *Spa*R were grown with an in meso approach^43^, similar to that used in our previous works^143,144^. Namely, the solubilized protein (40 mg/ml) in the crystallization buffer was mixed with premelted at 42 °C monoolein (MO, Nu-Chek Prep) in a 3:2 ratio (lipid:protein) to form a lipidic mesophase. The mesophase was homogenized in coupled syringes (Hamilton) by transferring the mesophase from one syringe to another until a homogeneous and gel-like material was formed. Then, 150 nl drops of a protein–mesophase mixture were spotted on a 96-well LCP glass sandwich plate (Marienfeld) and overlaid with 400 nL of precipitant solution by means of the NT8 crystallization robot (Formulatrix). The best crystals were obtained with a protein concentration of 20 mg/ml (in the water part of the mesophase). The crystals were obtained using 0.78 M NaH_2_PO_4_/K_2_HPO_4_ pH5.2 as a precipitant. The crystals were grown at 22 °C and appeared in 2 months. Once the crystals reached their final size, crystallization wells were opened, and drops containing the protein-mesophase mixture were covered with 100 μl of the respective precipitant solution. Before freezing, harvested crystals were incubated for 5 min in the respective precipitant solutions. Crystals were then harvested using micromounts (Mitegen, USA), flash-cooled and stored in liquid nitrogen.

### Diffraction data collection and treatment

X-ray diffraction data were collected at the ID29 beamline of ESRF (Grenoble, France) using a PILATUS 6M-F detector. Diffraction images were processed using XDS^145^. There is no possibility of twinning for the crystals. The data treatment statistics are presented in the Supplementary Table 1.

### Structure determination and refinement

Initial phases for the ground state of *Spa*R were successfully obtained in the C2 space group by molecular replacement using MOLREP^146^ from the CCP4 program suite^147^ using the 1C3W structure of BR rhodopsin^148^ as a search model. The initial models were iteratively refined using REFMAC5^149^ and Coot^150^. The structure refinement statistics are presented in the Supplementary Table 1.

## Supplementary information

Supplementary Information

Description of Additional Supplementary File

Supplementary Data 1

## Data Availability

Any relevant data are available from the authors upon reasonable request. Structural data of SpaR are deposited at the Protein Data Bank with accession number 8ANQ (also available as Supplementary Data 1).
