# Supplementary material for: Mirror proteorhodopsins
Source: Commun Chem. 2023 May 2;6:88. doi: 10.1038/s42004-023-00884-8 (PMC10154332; doi:10.1038/s42004-023-00884-8)
Supplement: Supplementary file 1 — Supplementary Information [file 42004_2023_884_MOESM1_ESM.pdf]

Supplementary Information

Mirror proteorhodopsins

Ivan S. Okhrimenko[1#], Kirill Kovalev[2#], Lada E. Petrovskaya[3], Nikolay S. Ilyinsky[1], Alexey A. Alekseev[1], Egor Marin[1+], Tatyana I. Rokitskaya[4], Yuri N. Antonenko[4], Sergey A. Siletsky[4], Petr A. Popov[1,5], Yuliya A. Zagryadskaya[1], Dmytro V. Soloviov[2], Igor V. Chizhov[6], Dmitrii V. Zabelskii[7], Yury L. Ryzhykau[1,8], Alexey V. Vlasov[1,8], Alexander I. Kuklin[1,8], Andrey O. Bogorodskiy[1], Anatolii E. Mikhailov[1], Daniil V. Sidorov[1], Siarhei Bukhalovich[1], Fedor Tsybrov[1], Sergey Bukhdruker[1], Anastasiia D. Vlasova[1], Valentin I. Borshchevskiy[1,8], Dmitry A. Dolgikh[3,9], Mikhail P. Kirpichnikov[3,9], Ernst Bamberg[10] and Valentin I. Gordeliy [11*]

[1] Research Center for Molecular Mechanisms of Aging and Age-related Diseases, Moscow Institute of Physics and Technology, Dolgoprudny, Russia

[2] European Molecular Biology Laboratory, Hamburg, Germany

[3] Shemyakin–Ovchinnikov Institute of Bioorganic Chemistry, RAS, Moscow, Russia

[4] Belozersky Institute of Physico-Chemical Biology, Lomonosov Moscow State University, Moscow, Russia

[5] iMolecule, Skolkovo Institute of Science and Technology, Moscow, Russia

[6] Institute for Biophysical Chemistry, Hannover Medical School, Hannover, Germany

[7] European XFEL, Schenefeld, Germany

[8] Frank Laboratory of Neutron Physics, Joint Institute for Nuclear Research, Dubna, Russia

[9] Biological Faculty, Lomonosov Moscow State University, Moscow, Russia

[10] Max Planck Institute of Biophysics, Frankfurt am Main, Germany

[11] Institut de Biologie Structurale (IBS), Université Grenoble Alpes, CNRS, CEA, Grenoble, France

[#] These authors contributed equally to the work

[+] Current affiliation: Groningen Biomolecular Sciences and Biotechnology Institute, University of Groningen, Groningen, The Netherlands

[*] Correspondence to: valentin.gordeliy@ibs.fr (V.G.)

Table 1. Data collection and refinement statistics.
Table 2. Parameters of liposomes with and without incorporated proteins obtained from SAXS data.
Figure 1. Sequence alignment of rhodopsins.
Figure 2. Sequence alignment of SpaR with related proteins.
Figure 3. Phylogenetic tree of microbial rhodopsins.
Figure 4. Phylogenetic tree of SpaR-like rhodopsins with host organisms.
Figure 5. Size-exclusion chromatography.
Figure 6. Fitting of the experimental SAXS data.
Figure 7. Spectral changes of SpaR in the presence and absence of Zn^{2+} as a function of pH.
Figure 8. Absolute absorption spectra of intermediates of the SpaR photocycle derived from the sequential irreversible scheme of relaxation.
Figure 9. pH dependence of the transient absorption changes of SpaR measured at 400 nm.
Figure 10. The pH dependence of half-time of re-protonation of the SpaR's Schiff base.
Figure 11. The pH changes induced by illumination.
Figure 12. SAXS data for the liposomes
Figure 13. Light-induced membrane potential changes ΔΨ in SpaR proteoliposomes.
Figure 14. Photocurrents of bR proteoliposomes adsorbed to a planar bilayer lipid membrane (BLM) at different pH and voltages.
Figure 15. Typical NG108-15 cells expressing SpaR-EYFP in their plasma membrane.
Figure 16. Comparison of the SpaR trimer with bR.
Figure 17. Structural alignment of SpaR with bR.
Figure 18. Comparison of overall protomer structures and cavities.
Figure 19. Detailed view of the extracellular part.
Figure 20. Detailed view of the cytoplasmic part.
Figure 21. Spectral comparison of DDM-solubilized S84D, S84E mutants and wild-type SpaR.
Figure 22. Pumping activity of SpaR and its mutants expressed in E. coli.
Figure 23. Traces of the transient absorption changes at 400 nm after photoexcitation of SpaR at different Zn^{2+} concentrations.
Figure 24. Zn^{2+} concentration dependence of amplitude of the absorption change of the slowest intermediate of the SpaR's.
Figure 25. Time traces of the absorption changes of SpaR at 400 nm measured at 10 mM of different divalent cations.
Figure 26. Melting temperature of SpaR in the presence and the absence of Zn^{2+} at pH5.5 and pH7.0.
Figure 27. Effect of calcium and magnesium ions on photocurrents of proteoliposomes with SpaR adsorbed to a planar bilayer lipid membrane (BLM).
Figure 28. SpaR optogenetically changes pH in lysosomes.

	SpaR
**Data collection**	
Space group	C2
Cell dimensions	
a, *b*, *c* (Å)	233.34, 65.03, 124.69
α, β, γ (°)	90, 90, 90
Resolution (Å)	46.32-2.80 (2.91-2.80)
R_{merge}	17.1 (124.5)
$I / \sigma I$	4.6 (0.7)
Completeness (%)	99.5 (97.3)
$CC_{1/2}$	99.5 (39.6)
Redundancy	2.3 (2.3)
**Refinement**	
Resolution (Å)	20.00-2.80
No. reflections	42,268
R_{work} / R_{free}	26.5/29.7
No. atoms	
Protein	10,234
Retinal	120
Water	33
Lipids	162
B-factors	
Protein	34
Retinal	33
Water	58
Lipids	70
R.m.s. deviations	
Bond lengths (Å)	0.007
Bond angles (°)	1.441

Supplementary Table 1 | Data collection and refinement statistics.

		Liposomes	Proteoliposomes
**Fit by form-factor of ULV with three-step electron density profile**	q-range, Å^{-1}	0.007 – 0.215	0.007 – 0.215
	Hydrophobic thickness T_{tail}, Å	35.5 ± 0.3	35.5 (fixed)
	Hydrophilic thickness T_{head}, Å	9 (fixed)	9 (fixed)
	$\Delta\rho_{tail} / \Delta\rho_{head}$	-0.237 ± 0.009	-0.192 ± 0.003
	Average vesicle radius R_{ULV}, Å	591 ± 10	305 ± 9
	Vesicle radius polidispersity σ/R, %	34 ± 2	35.6 ± 1.4
	Overall thickness* $T = T_{tail} + 2\ T_{head}$, Å	53.5 ± 0.3	53.5
	χ^2	1.4	1.1
**“Flat-particle” Guinier fit**	q-range, Å^{-1}	0.007 – 0.024	0.0165 – 0.034
	$q\,R_t$	0.22 – 0.75	0.56 – 1.16
	$q\,R_{ULV}$	4.14 – 14.18	5.03 – 10.37
	R_t, Å	31.2 ± 2.2	34.2 ± 1.0

* – deduced parameter.

Supplementary Table 2 | Parameters of liposomes with and without incorporated proteins obtained from SAXS data.

```
AER58218.2_ChR2_(C.reinhardtii)                      MASMAFSAISLASSAMRSLQASGGNPFEHDAPPDNSCELTPYGCLNDFYCNPAYGLADAG 60
Q9F7P4.1_PR_(Gamma-proteobacterium)                  ------------------------------------------------------------ 0
WP_011404249.1_XR_(S.ruber)                          ------------------------------------------------------------ 0
EGQ43296.1_NsXeR_(Nanosalina)                        ------------------------------------------------------------ 0
P42196_Sensory_rhodopsin-2_(N.pharaonis)             ------------------------------------------------------------ 0
WP_010903286.1_Sensory_rhodopsin-2_(H.salinarum)     ------------------------------------------------------------ 0
AAG01180.1_LR_(L.maculans)                           MIVDQFEEVLMKTSQLF--------PLPTATQSAQPTHVA-----------PVPTVLPDT 41
BAN14808.1_KR2_(D.eikasta)                           ------------------------------------------------------------ 0
WP_010902090.1_HR_(N.pharaonis)                      ------------------------------------MSIT-----------SVPGVVDAG 13
WP_012370306.1_ESR_(E.sibiricum)                     ------------------------------------------------------------ 0
P02945.2_BR_(H.salinarum)                            ------------------------------------------------------------ 0
WP_037567788.1_SpaR_(S.paucimobilis)                 ------------------------------------------------------------ 0


AER58218.2_ChR2_(C.reinhardtii)                      YNYCYVQSAYGKLAIVQTDQLSWLYSHG--------------SSGAKAASIAFQWLAFA- 105
Q9F7P4.1_PR_(Gamma-proteobacterium)                  ---------MKLLLI-----LGSVIALPT------FAAGGGDLD-ASDYTGVSFWLVTA- 38
WP_011404249.1_XR_(S.ruber)                          ----------------------MLQELPT------LTPGQYSL------VFNMFSFTVA- 25
EGQ43296.1_NsXeR_(Nanosalina)                        ----------------------MVYEAI--------------TAG--GFGSQPFILAYII 22
P42196_Sensory_rhodopsin-2_(N.pharaonis)             ------------------------------------------MVG----LTTLFWLGAI- 13
WP_010903286.1_Sensory_rhodopsin-2_(H.salinarum)     -------------------------------------------MA----LTTWFWVGAV- 12
AAG01180.1_LR_(L.maculans)                           ----------------------PIYE----------------TVG--DSGSKTLWVVFV- 60
BAN14808.1_KR2_(D.eikasta)                           ----------------------MTQELGNANFENFIGATEGFSEIAYQFTSHILTLGYA- 37
WP_010902090.1_HR_(N.pharaonis)                      VLGA--------------QSAAAVRE----------------NAL----LSSSLWVNVA- 38
WP_012370306.1_ESR_(E.sibiricum)                     ---------------------------------------MEEVNLLVLATQYMFWVGFV- 20
P02945.2_BR_(H.salinarum)                            -----------------------QAQ----------------ITG----RPEWIWLALGT 17
WP_037567788.1_SpaR_(S.paucimobilis)                 --------------------------------------------------MDWMAFLIGF 10
                                                                                                            .

AER58218.2_ChR2_(C.reinhardtii)                      -TAVIGLMFYAW-DTWKATTGWEEVYVCTIELIKVLIEIFK------------------- 144
Q9F7P4.1_PR_(Gamma-proteobacterium)                  -ALLASTVFFFV-ERDRVSAKWKTSLTVSGLVTGIAFWHYMYMRG--------------- 81
WP_011404249.1_XR_(S.ruber)                          -TMTASFVFFVL-ARNNVAPKYRISMMVSALVVFIAGYHYFRITSSWEAAYALQN----G 79
EGQ43296.1_NsXeR_(Nanosalina)                        TAMISG-LLFLYLPRKLDV--PQKFGIIHFFIVVWSGLMYTN------------------ 61
P42196_Sensory_rhodopsin-2_(N.pharaonis)             -GMLVGTLAFAWAG-RDAGSGERRYYVTLVGISGIAAVAYVVMALGVGWV------P--- 62
WP_010903286.1_Sensory_rhodopsin-2_(H.salinarum)     -GMLAGTVLPIR-D-CIRHPSHRRYDLVLAGITGLAAIAYTTMGLGITAT------T--- 60
AAG01180.1_LR_(L.maculans)                           -LMLIASAAFTALS-WKIPVNRRLYHVITTIITLTAALSYFAMATGHGVALN--KIVIRT 116
BAN14808.1_KR2_(D.eikasta)                           -VMLAGLLYFIL-TIKNVDKKFQMSNILSAVVMVSAFLLLYAQAQNWTSSFTFNEEVGRY 95
WP_010902090.1_HR_(N.pharaonis)                      -LAGIAILVFVYMGRTIRPGRPRLIWGATLMIPLVSISSYLGLLSGLTVGMI--EMPAG- 94
WP_012370306.1_ESR_(E.sibiricum)                     -GMAAGTLYFLV-ERNSLAPEYRSTATVAALVTFVAAIHYYFMKDAVGTS---------G 69
P02945.2_BR_(H.salinarum)                            ALMGLGTLYFLVKGMGVSDPDAKKFYAITTLVPAIAFTMYLSMLLGYGLTMV----P--- 70
WP_037567788.1_SpaR_(S.paucimobilis)                 TIMSLASLAIYAKGSKTSPAL--HHTLLHAAVPFIAATAYLAMTFGIGTL---------- 58
                                                          .                         :

AER58218.2_ChR2_(C.reinhardtii)                      EFEIPCSLYLPTGNWVLWLRYAEWLLTCPVILIHLSNITGLKDDYNK----RTMRLLVSD 200
Q9F7P4.1_PR_(Gamma-proteobacterium)                  VWI------ET-GDSPTVFRYIDWLLTVPLLICEFYLILAAATNV---AGSLFKKLLVGS 131
WP_011404249.1_XR_(S.ruber)                          MYQ------PTGELFNDAYRYVDWLLTVPLLTVELVLVMGLPKNE---RGPLAAKLGFLA 130
EGQ43296.1_NsXeR_(Nanosalina)                        --------FLNQSFLSDYAWYMDWMVSTPLILLALGLTAFHGADT--KRYDLLGALLGAE 111
P42196_Sensory_rhodopsin-2_(N.pharaonis)             --------V--AERTVFAPRYIDWILTTPLIVYFLGLLAGLDSRE-------FGIVITLN 105
WP_010903286.1_Sensory_rhodopsin-2_(H.salinarum)     --------V--GDRTVYLARYIDWLVTTPLIVLYLAMLARPGHRT-------SAWLLAAD 103
AAG01180.1_LR_(L.maculans)                           QHDHVPDTYETVYRQVYYARYIDWAITTPLLLLDLGLLAGMSGAH-------IFMAIVAD 169
BAN14808.1_KR2_(D.eikasta)                           FLD------PSGDLFNNGYRYLNWLIDVPMLLFQILFVVSLTTSK---FSSVRNQFWFSG 146
WP_010902090.1_HR_(N.pharaonis)                      ------HALAGEMVRSQWGRYLTWALSTPMILLALGLLADVDLGS-------LFTVIAAD 141
WP_012370306.1_ESR_(E.sibiricum)                     LLS------EI-DGFPTEIRYIDWLVTTPLLLVKFPLLLGLKGRL---GRPLLTKLVIAD 119
P02945.2_BR_(H.salinarum)                            --------FGGEQNPIYWARYADWLFTTPLLLLDLALLVDADQGT-------ILALVGAD 115
WP_037567788.1_SpaR_(S.paucimobilis)                 --------VNFNGSVTYLARYADWSVTTPILLASLVLLAFHERGRTGEVGGYLTAIIVLD 110
                                                                         *  * .  *::   :

AER58218.2_ChR2_(C.reinhardtii)                      IGCIVWGVTSAMTVGY------LKWIFFAIGLLYGSNTYFHSAKVYIEAYHTVPKGRC-R 253
Q9F7P4.1_PR_(Gamma-proteobacterium)                  LVMLVFGYMGEAGIMAA-------WPAFIIGCLAWVYMIYELWAGEGKSACNTASPAVQS 184
WP_011404249.1_XR_(S.ruber)                          ALMIVLGYPGEVSENAA-LFGTRGLWGFLS-TIPFVWILYILFTQLGDTIQRQSSRV-ST 187
EGQ43296.1_NsXeR_(Nanosalina)                        FTLVITGLLAQAQGSI---------TPYYVGVLLLLGVVYLLAKPFREIAEESSDGLA-R 161
P42196_Sensory_rhodopsin-2_(N.pharaonis)             TVVMLAGFAGAMVPG-----I-ERYALFGMGAVAFLGLVYYLVGPMTESASQRSSGIK-S 158
WP_010903286.1_Sensory_rhodopsin-2_(H.salinarum)     VFVIAAGIAAALTTG-----V-QRWLFFAVGAAGYAALLYGLLGTLPRALG-DDPRVR-S 155
AAG01180.1_LR_(L.maculans)                           LIMVLTGLFAAFGSEG----TPQKWGWYTIACIAYIFVVWHLVLNGGANARVKGEKLR-S 224
BAN14808.1_KR2_(D.eikasta)                           AMMIITGYIGQFYEVSN--LTAFLVWGAISSAFF--FHILWVMKKVINEGKEGISPAGQK 202
WP_010902090.1_HR_(N.pharaonis)                      IGMCVTGLAAAMTTSA----LLFRWAFYAISCAFFVVVLSALVTDWAASA--SSAGTA-E 194
WP_012370306.1_ESR_(E.sibiricum)                     VIMIVGGYIGESSINIAGGFTQLGLWSYLIGCFAWIYIIYLLFTNVTKAAENKPAPIR-D 178
P02945.2_BR_(H.salinarum)                            GIMIGTGLVGALTKV-----YSYRFVWWAISTAAMLYILYVLFFGFTSKAESMRPEVA-S 169
WP_037567788.1_SpaR_(S.paucimobilis)                 VLMIVTGLISSLAVV-----PALKWVWYLWSCAAFLGVLYLLWVPLRAMAVERGEALG-T 164
                                                           *  .

AER58218.2_ChR2_(C.reinhardtii)                      VIVRLMAYCFYLAWTMFPILFALG-----PEGMGQMSAY-MSTILTTIADVLSKQIWGLL 307
Q9F7P4.1_PR_(Gamma-proteobacterium)                  AYNT-MMYIIIFGWAIYPVGYFTGYLMGD------GGSALNLNLIYNLADFVNKILFGLI 237
WP_011404249.1_XR_(S.ruber)                          LLGN-ARLLLLATWGFYPIAYMIPMAFPEAFPSNTPGTIVALQVGYTIADVLAKAGYGVL 246
EGQ43296.1_NsXeR_(Nanosalina)                        AYKI-LAGYIGIFFLSYPTVWYISGIDALPGSLNILDPT-QTSIALVVLPFFCKQVYGFL 219
P42196_Sensory_rhodopsin-2_(N.pharaonis)             LYVR-LRNLTVILWAIYPFIWLLG-----PPGVALLTPT-VDVALIVYLDLVTKVGFGFI 211
WP_010903286.1_Sensory_rhodopsin-2_(H.salinarum)     LFVT-LRNITVVLWTLYPVVWLLS-----PAGIGILQTE-MYTIVVVYLDFISKVAFVAF 208
AAG01180.1_LR_(L.maculans)                           FFVA-IGAYTLILWTAYPIVWGLA-----DGARKIGVD--GEIIAYAVLDVLAKGVFGAW 276
BAN14808.1_KR2_(D.eikasta)                           ILSN-IWILFLISWTLYPGAYLMPYLTGVDGFLYSEDGVMARQLVYTIADVSSKVIYGVL 261
WP_010902090.1_HR_(N.pharaonis)                      IFDT-LRVLTVVLWLGYPIVWAVG-----VEGLALVQSVGVTSWAYSVLDVFAKYVFAFI 248
WP_012370306.1_ESR_(E.sibiricum)                     ALLK-MRLFILIGWAIYPIGYAVTLFAPG------VEIQLVRELIYNFADLTNKVGFGLI 231
P02945.2_BR_(H.salinarum)                            TFKV-LRNVTVVLWSAYPVVWLIG-----SEGAGIVPLN-IETLLFMVLDVSAKVGFGLI 222
WP_037567788.1_SpaR_(S.paucimobilis)                 AYQK-NVAFLTVIWFLYPIVFLIG-----PEGLKIISDP-TSVWAILIMDVLAKVVYAFY 217
                                                                  :  :*  :                             .  *  :

AER58218.2_ChR2_(C.reinhardtii)                      GHHLRVKIYQHILIHGDIRKKTTMQVGGEDV--EVEEFVDEDDEEGVRQANTQLANRESF 365
Q9F7P4.1_PR_(Gamma-proteobacterium)                  IWNVAVKESSNA------------------------------------------------ 249
WP_011404249.1_XR_(S.ruber)                          IYNIAKAKSEEEGFNVSEM-----------V--EP--ATASA------------------ 273
EGQ43296.1_NsXeR_(Nanosalina)                        DMYLIHKAE--------------------------------------------------- 228
P42196_Sensory_rhodopsin-2_(N.pharaonis)             ALDAAATLRAEHG---------ESLAGVDTD--AP--AVAD------------------- 239
WP_010903286.1_Sensory_rhodopsin-2_(H.salinarum)     AVLGADA-----V---------SRLVAADAA--AP--ATAEPTPDGD------------- 237
AAG01180.1_LR_(L.maculans)                           LLVTHANLRESDV---------ELN-GFWANGLNREGAIRIGEDDGA------------- 313
BAN14808.1_KR2_(D.eikasta)                           LGNLAITLSKNKELV------------------EA--NS--------------------- 280
WP_010902090.1_HR_(N.pharaonis)                      LLRWVANN--------------ERTVAVAGQTLGT--MSSDD------------------ 274
WP_012370306.1_ESR_(E.sibiricum)                     AFFAVKTMSSLSSSKGKTL-----------T--S-------------------------- 252
P02945.2_BR_(H.salinarum)                            LLRSRAIFGEAEA---------PEPSAGDGA---A--ATSD------------------- 249
WP_037567788.1_SpaR_(S.paucimobilis)                 AAANLETALRHHD---------VR---HDDR------------F---------------- 237
```

```
AER58218.2_ChR2_(C.reinhardtii)                         VHMAEQMKKNGIEVRATYDTGVDKEMGHHHVEAGRIILAVPDMSMVDFFRQQLSQMPAPI 425
Q9F7P4.1_PR_(Gamma-proteobacterium)                     ------------------------------------------------------------ 249
WP_011404249.1_XR_(S.ruber)                             ------------------------------------------------------------ 273
EGQ43296.1_NsXeR_(Nanosalina)                           ------------------------------------------------------------ 228
P42196_Sensory_rhodopsin-2_(N.pharaonis)                ------------------------------------------------------------ 239
WP_010903286.1_Sensory_rhodopsin-2_(H.salinarum)        ------------------------------------------------------------ 237
AAG01180.1_LR_(L.maculans)                              ------------------------------------------------------------ 313
BAN14808.1_KR2_(D.eikasta)                              ------------------------------------------------------------ 280
WP_010902090.1_HR_(N.pharaonis)                         ------------------------------------------------------------ 274
WP_012370306.1_ESR_(E.sibiricum)                        ------------------------------------------------------------ 252
P02945.2_BR_(H.salinarum)                               ------------------------------------------------------------ 249
WP_037567788.1_SpaR_(S.paucimobilis)                    ------------------------------------------------------------ 237


AER58218.2_ChR2_(C.reinhardtii)                         ELVPALGIDNTVQLVQQAAALGGCDFVLVHPEFLKDASSSGLVTKLRMMGQRVCAFGWSP 485
Q9F7P4.1_PR_(Gamma-proteobacterium)                     ------------------------------------------------------------ 249
WP_011404249.1_XR_(S.ruber)                             ------------------------------------------------------------ 273
EGQ43296.1_NsXeR_(Nanosalina)                           ------------------------------------------------------------ 228
P42196_Sensory_rhodopsin-2_(N.pharaonis)                ------------------------------------------------------------ 239
WP_010903286.1_Sensory_rhodopsin-2_(H.salinarum)        ------------------------------------------------------------ 237
AAG01180.1_LR_(L.maculans)                              ------------------------------------------------------------ 313
BAN14808.1_KR2_(D.eikasta)                              ------------------------------------------------------------ 280
WP_010902090.1_HR_(N.pharaonis)                         ------------------------------------------------------------ 274
WP_012370306.1_ESR_(E.sibiricum)                        ------------------------------------------------------------ 252
P02945.2_BR_(H.salinarum)                               ------------------------------------------------------------ 249
WP_037567788.1_SpaR_(S.paucimobilis)                    ------------------------------------------------------------ 237


AER58218.2_ChR2_(C.reinhardtii)                         MGPQRELIESRGLDGWLEGPSFGSGIDRHQLTALVSRMQMMRKATMGSGMANPMAQQQQS 545
Q9F7P4.1_PR_(Gamma-proteobacterium)                     ------------------------------------------------------------ 249
WP_011404249.1_XR_(S.ruber)                             ------------------------------------------------------------ 273
EGQ43296.1_NsXeR_(Nanosalina)                           ------------------------------------------------------------ 228
P42196_Sensory_rhodopsin-2_(N.pharaonis)                ------------------------------------------------------------ 239
WP_010903286.1_Sensory_rhodopsin-2_(H.salinarum)        ------------------------------------------------------------ 237
AAG01180.1_LR_(L.maculans)                              ------------------------------------------------------------ 313
BAN14808.1_KR2_(D.eikasta)                              ------------------------------------------------------------ 280
WP_010902090.1_HR_(N.pharaonis)                         ------------------------------------------------------------ 274
WP_012370306.1_ESR_(E.sibiricum)                        ------------------------------------------------------------ 252
P02945.2_BR_(H.salinarum)                               ------------------------------------------------------------ 249
WP_037567788.1_SpaR_(S.paucimobilis)                    ------------------------------------------------------------ 237


AER58218.2_ChR2_(C.reinhardtii)                         FMMHQQNSAHNSFMIQPQTQPQANPLYGAQMGSQMGATTGSALFHPAAPGNATPPSPSGA 605
Q9F7P4.1_PR_(Gamma-proteobacterium)                     ------------------------------------------------------------ 249
WP_011404249.1_XR_(S.ruber)                             ------------------------------------------------------------ 273
EGQ43296.1_NsXeR_(Nanosalina)                           ------------------------------------------------------------ 228
P42196_Sensory_rhodopsin-2_(N.pharaonis)                ------------------------------------------------------------ 239
WP_010903286.1_Sensory_rhodopsin-2_(H.salinarum)        ------------------------------------------------------------ 237
AAG01180.1_LR_(L.maculans)                              ------------------------------------------------------------ 313
BAN14808.1_KR2_(D.eikasta)                              ------------------------------------------------------------ 280
WP_010902090.1_HR_(N.pharaonis)                         ------------------------------------------------------------ 274
WP_012370306.1_ESR_(E.sibiricum)                        ------------------------------------------------------------ 252
P02945.2_BR_(H.salinarum)                               ------------------------------------------------------------ 249
WP_037567788.1_SpaR_(S.paucimobilis)                    ------------------------------------------------------------ 237


AER58218.2_ChR2_(C.reinhardtii)                         ANVNEAEMLQQLMGEITRLKSELGGSGTPR     635
Q9F7P4.1_PR_(Gamma-proteobacterium)                     ------------------------------     249
WP_011404249.1_XR_(S.ruber)                             ------------------------------     273
EGQ43296.1_NsXeR_(Nanosalina)                           ------------------------------     228
P42196_Sensory_rhodopsin-2_(N.pharaonis)                ------------------------------     239
WP_010903286.1_Sensory_rhodopsin-2_(H.salinarum)        ------------------------------     237
AAG01180.1_LR_(L.maculans)                              ------------------------------     313
BAN14808.1_KR2_(D.eikasta)                              ------------------------------     280
WP_010902090.1_HR_(N.pharaonis)                         ------------------------------     274
WP_012370306.1_ESR_(E.sibiricum)                        ------------------------------     252
P02945.2_BR_(H.salinarum)                               ------------------------------     249
WP_037567788.1_SpaR_(S.paucimobilis)                    ------------------------------     237
```

Supplementary Figure 1 | Sequence alignment of rhodopsins. The sequence alignment was performed with Clustal Omega. The SpaR sequence is compared to the best-studied rhodopsins. The motif amino acids are coloured blue. Highly conserved regions are coloured red.

```
EST15450.1_PspR_(P.putida)                    ----------MIQTPLLIGFIVMALASLAIYIKGA-HYGPLLGHTLIHAAVPFIAATAYL      49
ADD77066.1_PaR_(P.ananatis)                   MRASVQGESIMDQTAFLIGFSVMALASLVIYASGK-KIFPLRHHTLMHASVPFIAATAYL      59
ADO10039.1_PvR_(P.vagans)                     ----------MDQTAFMIGFSVMAIASLIIYATGD-KKYPFGHHTLVHASVPFIAATAYL      49
WP_010215292.1_SpR_(Sphingomonas_PAMC26621)   ----------MDQTAFLVGFTVMSLGSLAIYATGK-KSCPAGHHTLLHASVPFIAATAYL      49
WP_025840800.1_ApR_(A.platycodi)              ----------MTLTVLWVTALIMFAGGTALLLLGKRRTETEGVMSLAHGIVPIIAGCLYV      50
WP_010997316.1_ASR_(Anabaena)                 --------MNLESLLHWIYVAGMTIGALHFWSLSRNPRGVPQYEYLVAMFIPIWSGLAYM      52
WP_037567788.1_SpaR_(S.paucimobilis)          ----------MDWMAFLIGFTIMSLASLAIYAKGS-KTSPALHHTLLHAAVPFIAATAYL      49
EGI56673.1_(Sphingomonas_S17)                 -------------MAFLIGFTIMSLASLAIYAKGS-KTSPALHHTLLHAAVPFIAATAYL      46
WP_076714729.1_(Sphingomonas_LK11)            ----------MDWMAFLIGFTIMSLASLAIYAKGS-KTSPSLHHTLLHAAVPFIAATAYL      49
WP_058744770.1_(S.yabuuchiae)                 ----------MDWMAFLIGFTIMSLASLAIYAKGS-KTSPSLHHTLLHAAVPFIAATAYL      49
WP_056431997.1_(Sphingomonas_Leaf257)         ----------MDWMAFLIGFTIMSLASLAIYAKGS-KTSPSLHHTLLHAAVPFIAATAYL      49
WP_110152629.1_(Nostoc_3335mG)                ----------MDQTAFMIGFTVMSLASLAIYAKGE-KTPPSHHHTLLHAAVPFIAATAYL      49
WP_037523293.1_(Sphingomonas_Mn802worker)     ----------MDGMVFLIGFTIMSLGSLAIYATGS-KEPPSRHHTLLHASVPFIAATAYL      49
WP_093007023.1_(Sphingomonas_JS21-1)          ----------MDGTAFLIGFTILSLGSLAIYATGS-KQPPSRHHTLLHASVPFIAATAYL      49
WP_093333672.1_(S.rubra)                      ----------MDQTAFMIGFTVMSLASLAIYAKGG-KSPPSRHHTLLHAAVPFMAASAYL      49
WP_038658594.1_(S.taxi)                       ----------MDQTAFLLGFTIMSLGSLAIYATGK-KSHPSGHHTLLHASVPFIAATAYL      49
                                                               :    :  ..  :   .           *    :*: :.  *:

EST15450.1_PspR_(P.putida)                    CMYLGVGNLIKVD----------GSVTYLARYVDWAFTTPLLLAGVVSSAYYGTRDLYGK      99
ADD77066.1_PaR_(P.ananatis)                   AMAFGFGNLATES----------GSIVYLARYADWSVTTPVLLAGLVMVAFHEQSKPGEM      109
ADO10039.1_PvR_(P.vagans)                     AMAFGLGNLTLDN----------GTVVYLARYADWSVTTPLLLAGLVMLAFHEQGKPGEM      99
WP_010215292.1_SpR_(Sphingomonas_PAMC26621)   AMAFGIGTLLSFD----------GSITYLARYLDWSVTTPILLAGLALTAFHEHGKPGTV      99
WP_025840800.1_ApR_(A.platycodi)              AMATGQGALLLPTDATLAGAANTTRIFWFGRYIDWLFTTPLLLVGLGFLGMFRGRKR---      107
WP_010997316.1_ASR_(Anabaena)                 AMAIDQGKVEAA-----------GQIAHYARYIDWMVTTPLLLLSLSWTAMQFIKKDWTL      101
WP_037567788.1_SpaR_(S.paucimobilis)          AMTFGIGTLVNFN----------GSVTYLARYADWSVTTPILLASLVLLAFHERGRTGEV      99
EGI56673.1_(Sphingomonas_S17)                 AMTFGIGTLVNFN----------GSVTYLARYADWSVTTPILLASLVLLAFHERGRTGEV      96
WP_076714729.1_(Sphingomonas_LK11)            AMAFGIGTLINID----------GSVTYLARYADWSVTTPILLASLVLLAFHERGKMGEV      99
WP_058744770.1_(S.yabuuchiae)                 AMAFGIGTLINID----------GSVTYLARYADWSVTTPILLASLVLLAFHERGKTGEV      99
WP_056431997.1_(Sphingomonas_Leaf257)         AMAFGIGTLINID----------GSVTYLARYADWSVTTPILLASLVLLAFHERGKTGEV      99
WP_110152629.1_(Nostoc_3335mG)                AMAFGIGTVVKGD----------GSATYFARYADWTVTTPILLSSLVLLAFHERGKTGEV      99
WP_037523293.1_(Sphingomonas_Mn802worker)     AMAFGIGTLVKLD----------GSATYLARYLDWSVTTPILLAGLVLVAFHERGSTGEV      99
WP_093007023.1_(Sphingomonas_JS21-1)          AMAFGIGTLVKFD----------GTATYFARYADWSVTTPILLASLVLLAFHERGSTGEV      99
WP_093333672.1_(S.rubra)                      AMAFGIGTLVKVD----------GSATYFARYIDWSVTTPILLSSLVLLAFHERGKTGEV      99
WP_038658594.1_(S.taxi)                       AMAFGIGTLFKLD----------GSVTYLARYLDWSVTTPILLSSLVLLAFHEHGKTGEV      99
                                              .*  . * :                    .** ** .***:** .:   .

EST15450.1_PspR_(P.putida)                    SGYITAIVTLDVIMIVTGLIASLAPYGVIKWVFFAWSCAAFAGVLYLLWKPVASIASQ-Q      158
ADD77066.1_PaR_(P.ananatis)                   GGFLTAIIILDVMMIITGLVSALATTFAAKWVWYLWSCAAFLGVVYLLWGPLRTIAAGRG      169
ADO10039.1_PvR_(P.vagans)                     GGFLTAIIVLDVMMIITGLVSSLAETSVAKWVWYLWSCAAFLGVVYLLWGPLRAIAATRG      159
WP_010215292.1_SpR_(Sphingomonas_PAMC26621)   GGYLTAIIVLDVLMILTGLISSLAPASPAKWVWYVWSCVAFVGVLYLLWGPLLKHARSHS      159
WP_025840800.1_ApR_(A.platycodi)              TDLLLGAVAADVIMVITAFAFSASENLVDRWIWFILSCVAFLGVYYVIWVSQLQANRLER      167
WP_010997316.1_ASR_(Anabaena)                 IGFLMS---TQIVVITSGLIADLSERDWVRYLWYICGVCAFLIILWGIWNPLRAKTRTQS      158
WP_037567788.1_SpaR_(S.paucimobilis)          GGYLTAIIVLDVLMIVTGLISSLAVVPALKWVWYLWSCAAFLGVLYLLWVPLRAMAVERG      159
EGI56673.1_(Sphingomonas_S17)                 GGYLTAIIVLDVLMIVTGLISSLAVVPALKWVWYLWSCAAFLGVLYLLWVPLRAMAVERG      156
WP_076714729.1_(Sphingomonas_LK11)            GGYLTAIIVLDVLMIVTGLISSLALVPVLKWVWYLWSCAAFVGVLYLLWVPLRAMAAERG      159
WP_058744770.1_(S.yabuuchiae)                 GGYLTAIIVLDVLMIVTGLISSLALVPVLKWVWYLWSCAAFVGVLYLLWGPLRVLAAERG      159
WP_056431997.1_(Sphingomonas_Leaf257)         GGYLTAIIVLDVLMIVTGLISSLALVPVLKWVWYLWSCAAFVGVLYLLWGPLRVLAAERG      159
WP_110152629.1_(Nostoc_3335mG)                GGYLTAIIVLDVLMIETGLISSLAEAPLTKWVWYAWSCAAFLGVLYLLWGPLRAMAVERG      159
WP_037523293.1_(Sphingomonas_Mn802worker)     GGYLTAIIVLDVLMIVTGLISSLAIVPVGKWVWYLWSCVAFAGVLYLLWVPLRARAFERG      159
WP_093007023.1_(Sphingomonas_JS21-1)          GGYLTSIIVLDVLMILTGLISSLAAVPVIKWVWYLWSCVAFAGVLYLLWVPLRARAMERG      159
WP_093333672.1_(S.rubra)                      GGYLTSIIVLDVLMIATGLIASLAEVPLVKWVWYLWSCAAFAGVLYLLWGPLRTMAAERG      159
WP_038658594.1_(S.taxi)                       GGHLTAIIVLDVLMIVTGLVSSLAGPSFAKWVWYAWSCVAFLGVLYLLWGPLRTLAQSRG      159
                                               . : .    ::::: :.:    :     :::::  .  **  : : :*

EST15450.1_PspR_(P.putida)                    PGVSPAYRRNVGFLTVLWLIYPVVFAVGPEGFWAVSDATTVWVFLVLDVLAKVVYAFTSE      218
ADD77066.1_PaR_(P.ananatis)                   RAMAVAYNKNVALLTVVWFIYPIVFLVGPEGLKIITDPASVWAFLVLDIIAKVFYAFYAA      229
ADO10039.1_PvR_(P.vagans)                     NALAGAYNKNVALLTIVWFIYPIVFLVGPEGLRIITDATSVWAFLILDIIAKVIYAFYAA      219
WP_010215292.1_SpR_(Sphingomonas_PAMC26621)   AALGTAYTKNLTFLTVVWFLYPIVFLLGPEGLKVITDPTSVWAFLIMDIVAKVVYAFYAA      219
WP_025840800.1_ApR_(A.platycodi)              PEVQSAYIRSAAILSVLWMVYPVILALSPDGLGLVSDAASVLIIAVTDVLAKVGFGFLGL      227
WP_010997316.1_ASR_(Anabaena)                 SELANLYDKLVTYFTVLWIGYPIVWIIGPSGFGWINQTIDTFLFCLLPFFSKVGFSFLDL      218
WP_037567788.1_SpaR_(S.paucimobilis)          EALGTAYQKNVAFLTVIWFLYPIVFLIGPEGLKIISDPTSVWAILIMDVLAKVVYAFYAA      219
EGI56673.1_(Sphingomonas_S17)                 EALGTAYQKNVAFLTVIWFLYPIVFLIGPEGLKIISDPTSVWAILIMDVLAKVVYAFYAA      216
WP_076714729.1_(Sphingomonas_LK11)            EALGTAYRKNVVFLTVIWFLYPIVFLVGPEGLKIISDPTSVWAILIMDVLAKVVYAFYAA      219
WP_058744770.1_(S.yabuuchiae)                 EALGTAYRKNVVFLTVIWFLYPIVFLVGPEGLKIISDPMSVWAILIMDVLAKVVYAFYAA      219
WP_056431997.1_(Sphingomonas_Leaf257)         EALGTAYRKNVVFLTVIWFLYPIVFLVGPEGLKIISDPTSVWAILIMDVLAKVVYAFYAA      219
WP_110152629.1_(Nostoc_3335mG)                SALGEAYRKNVTFLTVIWFLYPIVFLIGPEGLKIITDPASVWAILIMDILAKVVYAFYAA      219
WP_037523293.1_(Sphingomonas_Mn802worker)     QALGSAYVKNVGFLTVIWFLYPIVFLVGPEGLKIVTDLTTVWAILIMDVVAKVVYAFYAA      219
WP_093007023.1_(Sphingomonas_JS21-1)          PALGSAYLKNVGFLTVVWFLYPIVFLIGPEGLKIINDPTSVWAILIMDVVAKVVYAFYAA      219
WP_093333672.1_(S.rubra)                      GALGDAYGKNVTFLTVVWFLYPIVFLVGPEGLKIISDPASVWAILVMDIVAKVAYAFYAA      219
WP_038658594.1_(S.taxi)                       MALGTAYNKNVAFLTVIWFLYPVVFLIGPEGMKIITDPASVWAILIMDVVAKIVYAFYAA      219
                                                :   * :    ::::*: **::  :.*.*:  :.:   .  : :  ..:*: :.*

EST15450.1_PspR_(P.putida)                    RNLRAVPVGRG----Y-------------------------          230
ADD77066.1_PaR_(P.ananatis)                   SSLEKALRHSTSDGAVRV-----------------------          247
ADO10039.1_PvR_(P.vagans)                     ANMKKALSHAVPVGAVRR-----------------------          237
WP_010215292.1_SpR_(Sphingomonas_PAMC26621)   VNLDKALPHQGDDRPYRS-----------------------          237
WP_025840800.1_ApR_(A.platycodi)              SDDRELTTV--------------------------------          236
WP_010997316.1_ASR_(Anabaena)                 HGLRNLNDSRQTTGDRFAENTLQFVENITLFANSRRQQSRRRV        261
WP_037567788.1_SpaR_(S.paucimobilis)          ANLETALRHHDVRHDDRF-----------------------          237
EGI56673.1_(Sphingomonas_S17)                 ANLETALRHHDVRHDDRF-----------------------          234
WP_076714729.1_(Sphingomonas_LK11)            ANLKTALHDHRA-----------------------------          231
WP_058744770.1_(S.yabuuchiae)                 ANLKTALHDHRA-----------------------------          231
WP_056431997.1_(Sphingomonas_Leaf257)         ANLKTALHDHRA-----------------------------          231
WP_110152629.1_(Nostoc_3335mG)                GNLEKALHEHARQA---------------------------          233
WP_037523293.1_(Sphingomonas_Mn802worker)     NNVEKALRERGSG--YEHDRVH----A--------------          240
WP_093007023.1_(Sphingomonas_JS21-1)          NNVDKALRERGSDHGYEHGHEHGRVHA--------------          246
WP_093333672.1_(S.rubra)                      SNLDKALHQHGSDGGYRA-----------------------          237
WP_038658594.1_(S.taxi)                       SNLDKALEQHNTERGHQS-----------------------          237
```

Supplementary Figure 2 | Sequence alignment of SpaR with related proteins. SpaR is compared to rhodopsins with the RDTGDK and RDTSDK motif. *Anabaena* sensory rhodopsin (ASR) is shown too. The sequence alignment was performed with Clustal Omega. The motif amino acids are coloured blue. Highly conserved regions are colored red. A unique conserved His residue is highlighted yellow.

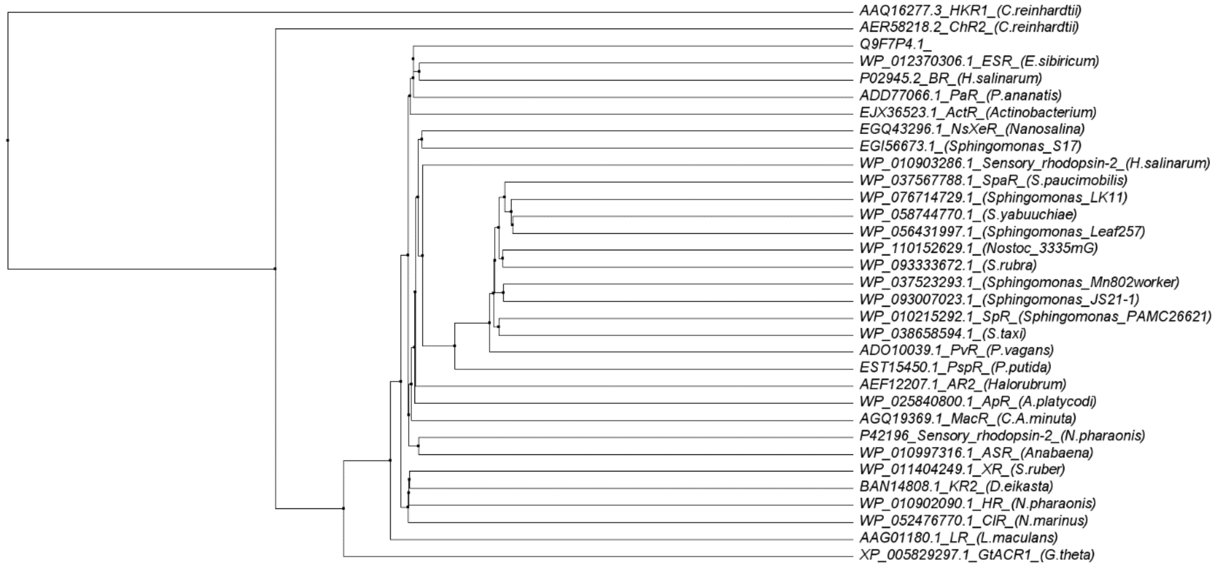

Supplementary Figure 3 | Phylogenetic tree of microbial rhodopsins. Thirty-three rhodopsin sequences were aligned using MUSCLE. The phylogenetic tree was built using Jalview 2. The Genbank accession numbers are written in parentheses for each protein.

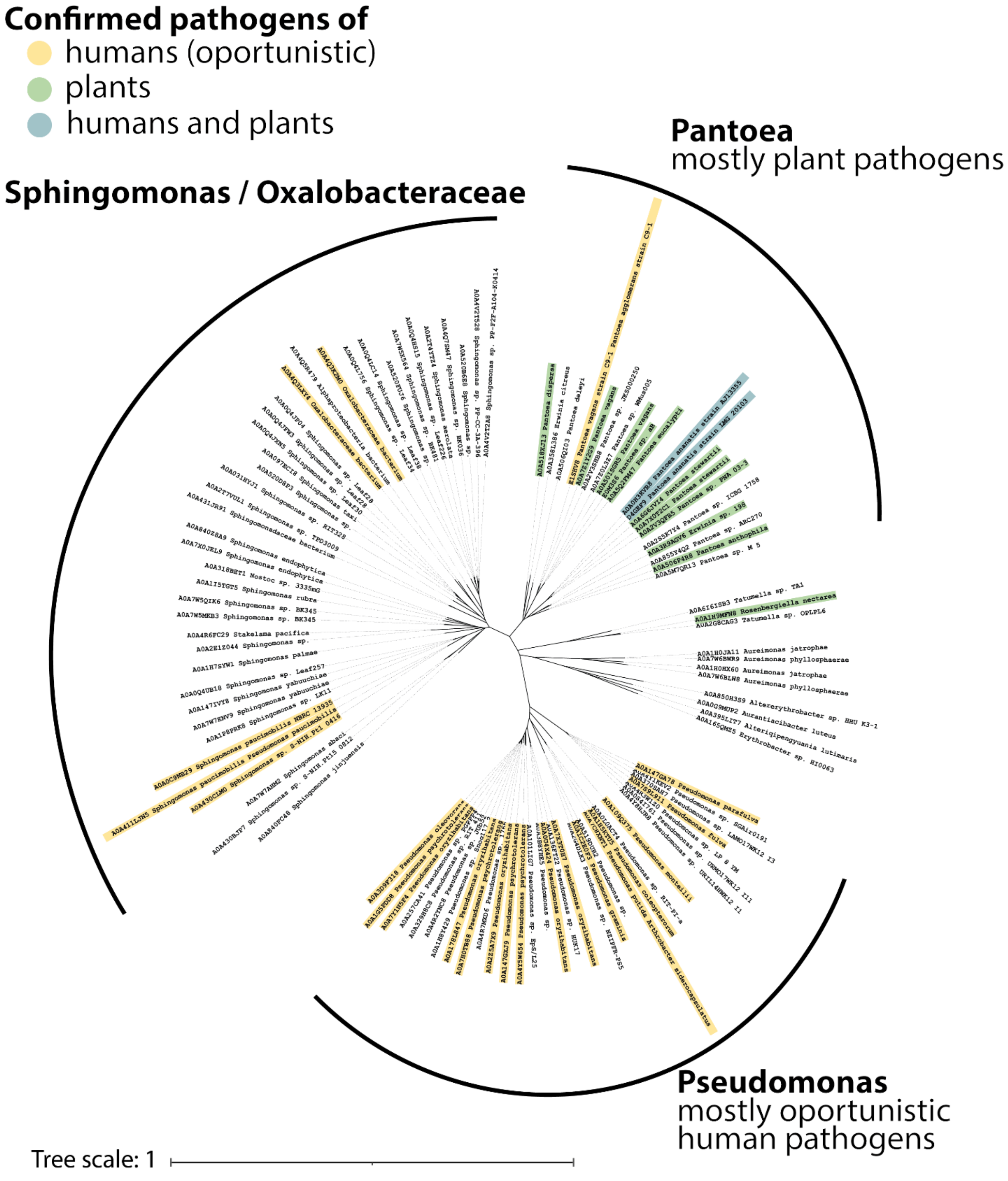

Supplementary Figure 4 | Phylogenetic tree of SpaR-like rhodopsins with host organisms. All organisms, which are confirmed pathogens of humans or plants, are highlighted with a specific colour according to the legend.

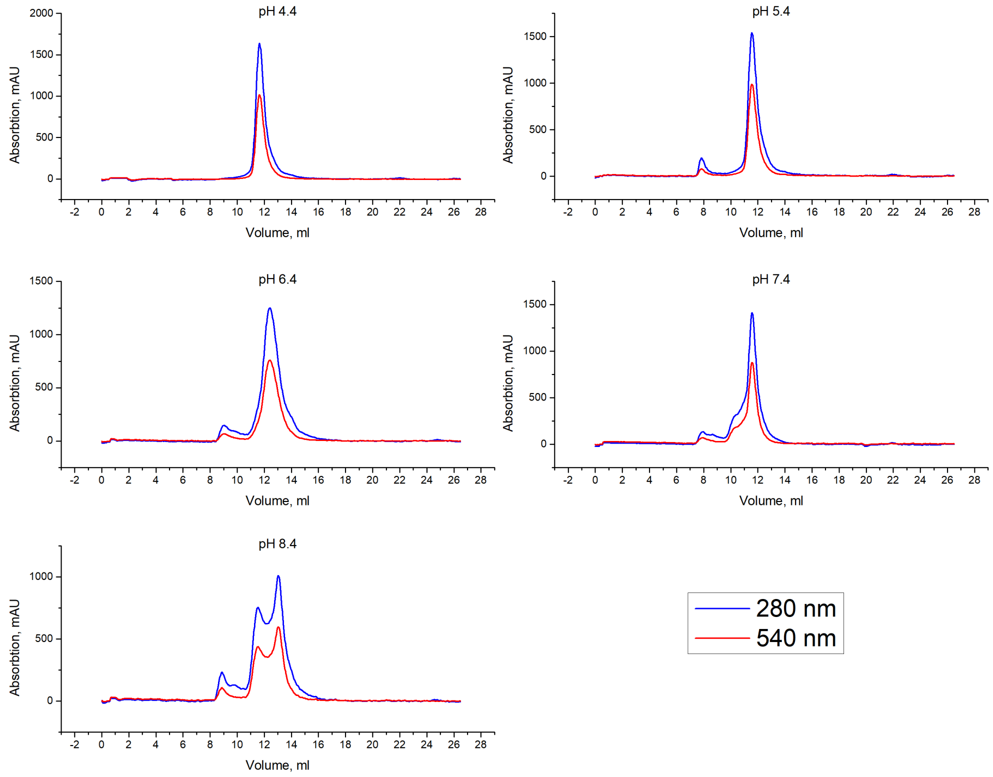

Supplementary Figure 5 | Size-exclusion chromatography. The size-exclusion chromatograms of SpaR protein at different pH (50 mM sodium phosphate, 100 mM NaCl, +4°C) measured at 280 nm (blue lines) and 540 nm (red lines).

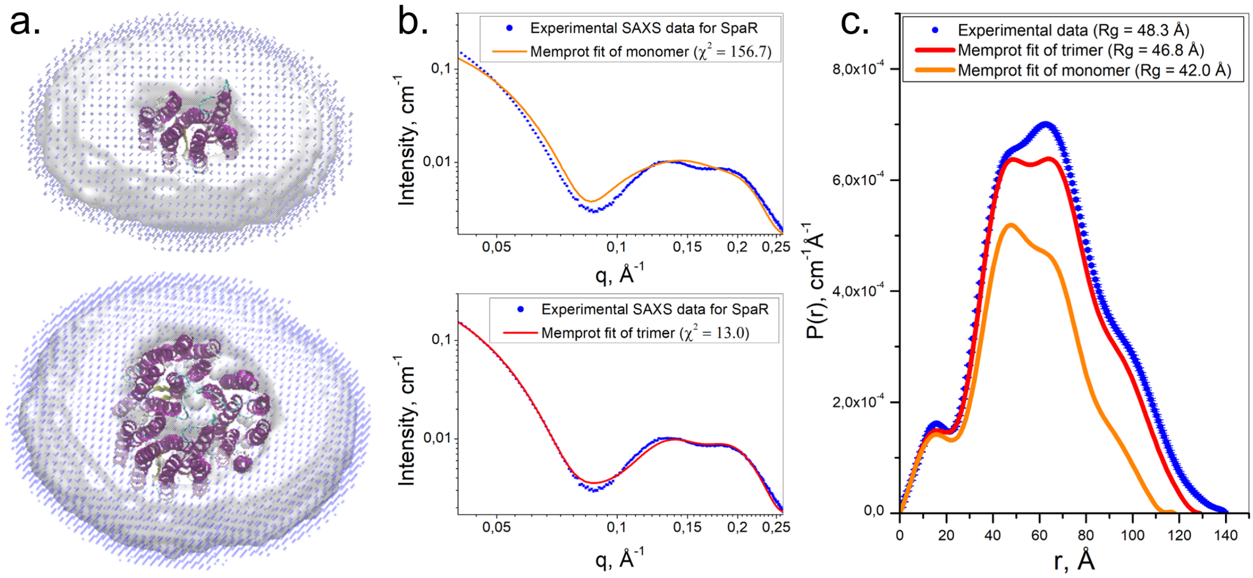

Supplementary Figure 6 | Fitting of the experimental SAXS data. The data show that the trimer of SpaR fits the SAXS data. **a)** MEMPROT best models for monomer and trimer of the SpaR with detergent corona. **b)** Fits of the experimental SAXS data obtained with monomeric and trimeric SpaR models. **c)** P(r) calculated for the experimental data and the monomeric and trimeric MEMPROT fits.

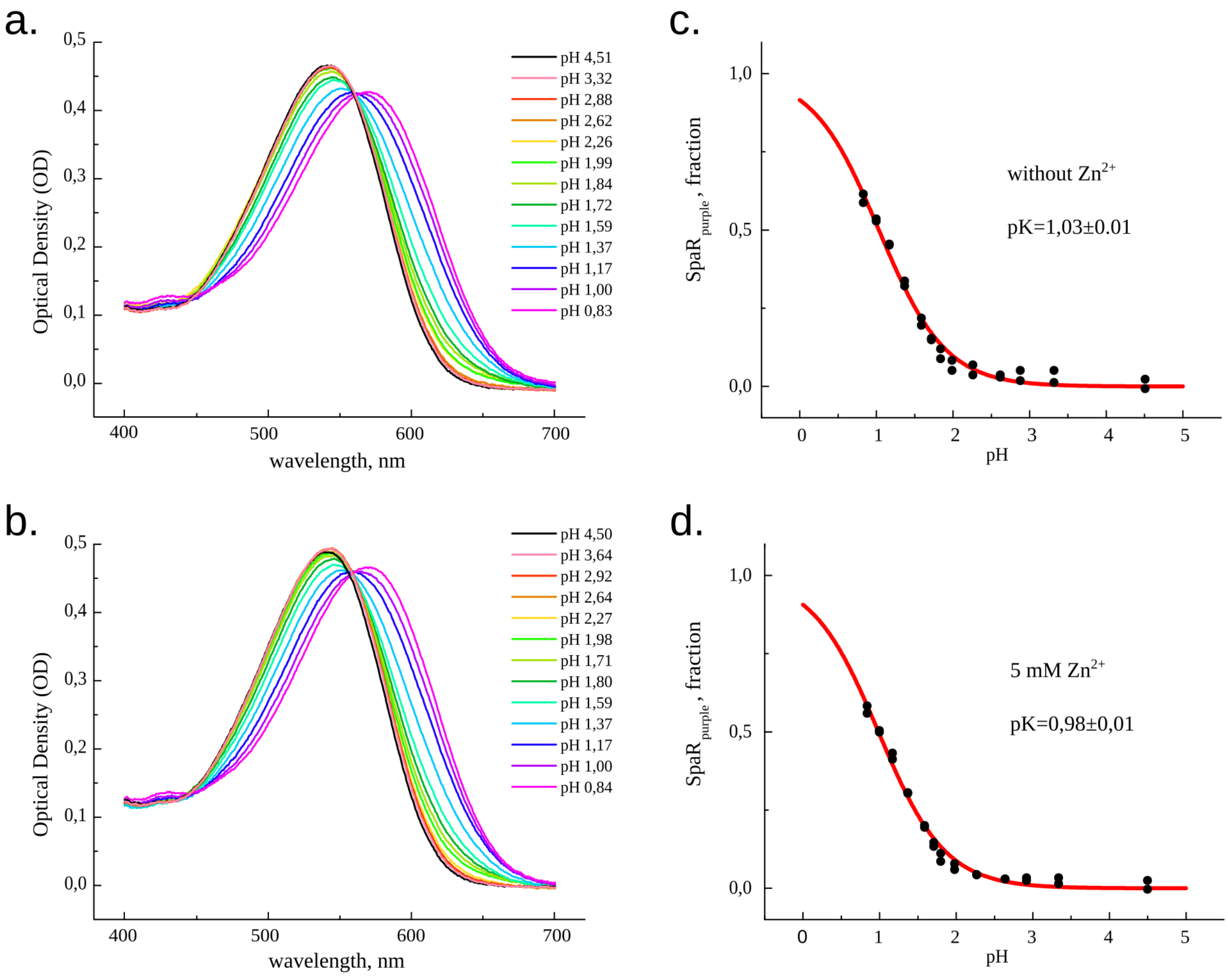

Supplementary Figure 7 | Spectral changes of SpaR in the presence and absence of Zn^{2+} as a function of pH. The maximum of absorption of retinal Schiff base (RSB) of SpaR spectrum is shifted from 540 nm to 570 nm (pink and purple states respectively) during the lowering the pH. Measurements were done in 50 mM MES, 150 mM NaCl, 0,05% DDM, **a**) 0 or **b**) 5 mM of $ZnCl_2$. The spectral changes are reversible, but slight denaturation of SpaR at low pH is observed during incubation of the protein. The maximum changes of OD in response of change of pH were observed at 515 nm and 615 nm, global fitting of OD at these wavelengths by equation $OD=A_0+A_1\frac{10^{-pH}}{10^{-pH}+10^{-pK}}$ was done, then the results were normalized and shown as fractions of protonated states on right panels (**c** and **d**). Fractions of purple state of SpaR at each pH are presented by the black circles. The solid red curve depicts these data theoretical fit giving pK=1.03±0.01 and 0.98±0.01 for SpaR in the absence and presence of Zn^{2+} (**c** and **d** respectively). These values reflects the pK_{D73} of proton acceptor group Asp73, which corresponds to Asp85 Halobacterium salinarum bacteriorhodopsin and is close to RSB. The pK_{D73} is notably low and do negligible depends on Zn^{2+} concentration.

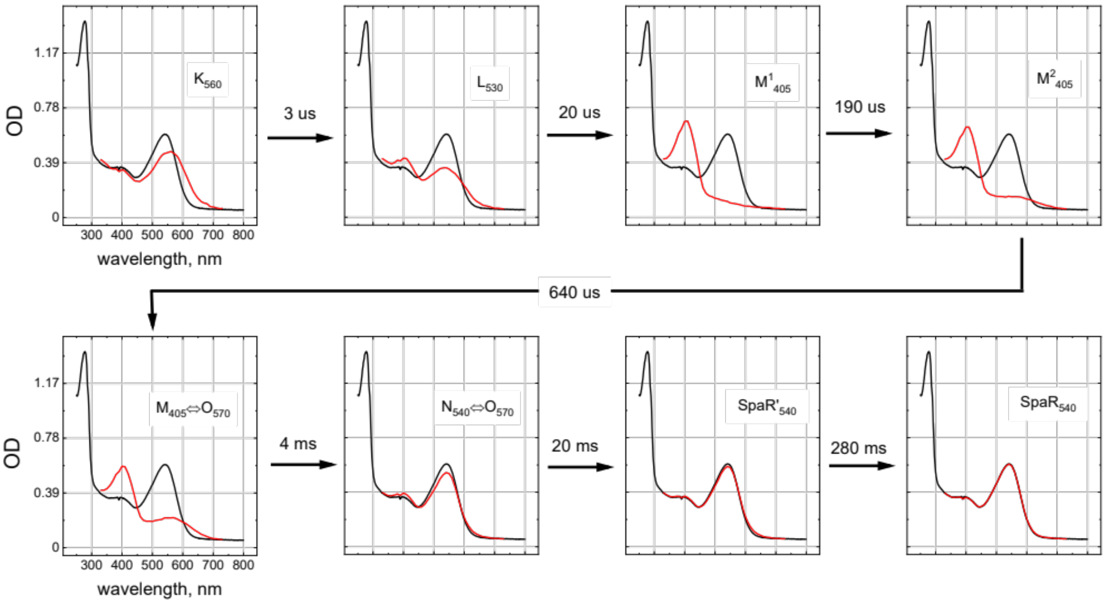

Supplementary Figure 8 | Absolute absorption spectra of intermediates of the SpaR photocycle derived from the sequential irreversible scheme of relaxation. The half-times of the reactions corresponds to 20°C and pH 5.0.

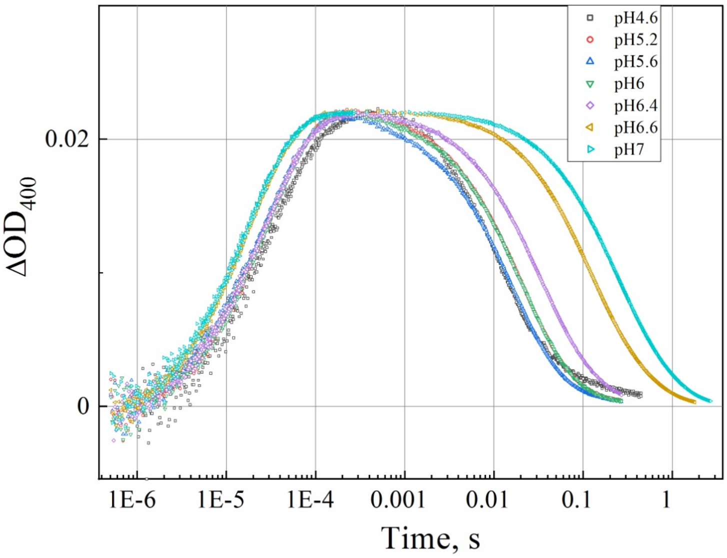

Supplementary Figure 9 | pH dependence of the transient absorption changes of SpaR measured at 400 nm. Traces of the transient absorption changes at 400 nm after photoexcitation of SpaR in time at different pH (4.6, 5.2, 5.6, 6.0, 6.4, 6.6, 7.0) are shown by different colours according the legend.

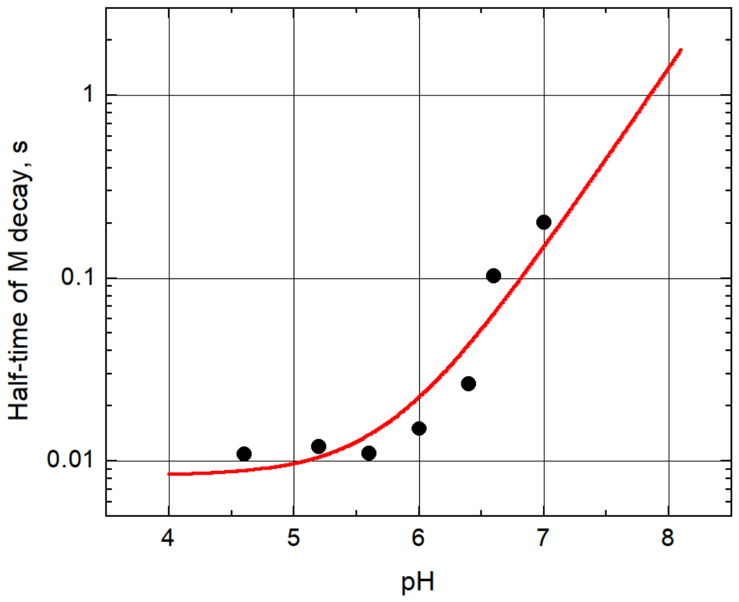

Supplementary Figure 10 | The pH dependence of half-time of re-protonation of the SpaR's Schiff base. The data points (black cycles) were obtained from fig.3 using single exponential approximation of the transient absorption decay at 400 nm at different pHs. The solid red curve depicts the data theoretical fit. The function $\tau_{1/2} = \tau_0 \frac{10^{-pK}+10^{-pH}}{10^{-pH}}$ was been used for the data fit. This formula describes the influence of protonation of the presumable proton donor group (corresponds to Asp96 in *Halobacterium salinarum* bacteriorhodopsin) on the apparent half-time of the Schiff base re-protonation. The red curve is the best fit of the data giving pK=5.8±0.2 and τ_0 =8±3 ms.

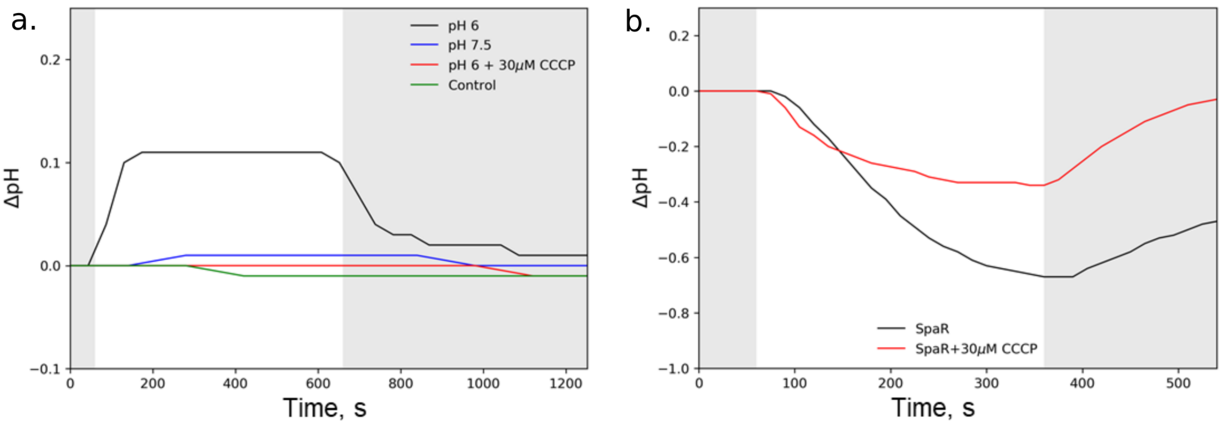

Supplementary Figure 11 | The pH changes induced by illumination. The illumination causes changes of pH in the suspension **a)** of the lipid vesicles with reconstituted SpaR and the **b)** *E. coli* cells expressing SpaR. White area shows illumination time period, grey areas indicate periods when light is off. **a)** Lipid vesicles with reconstituted SpaR were used in suspension in solutions (100 mM sodium chloride) with initial pH equal to 6,0 (without and with 30 µM CCCP) and 7,5. Single lipid vesicles without SpaR protein were used as control. **b)** The *E. coli* cells expressing the SpaR were used in suspension in unbuffered solution of 100 mM NaCl without and with thee addition of 30 µM CCCP.

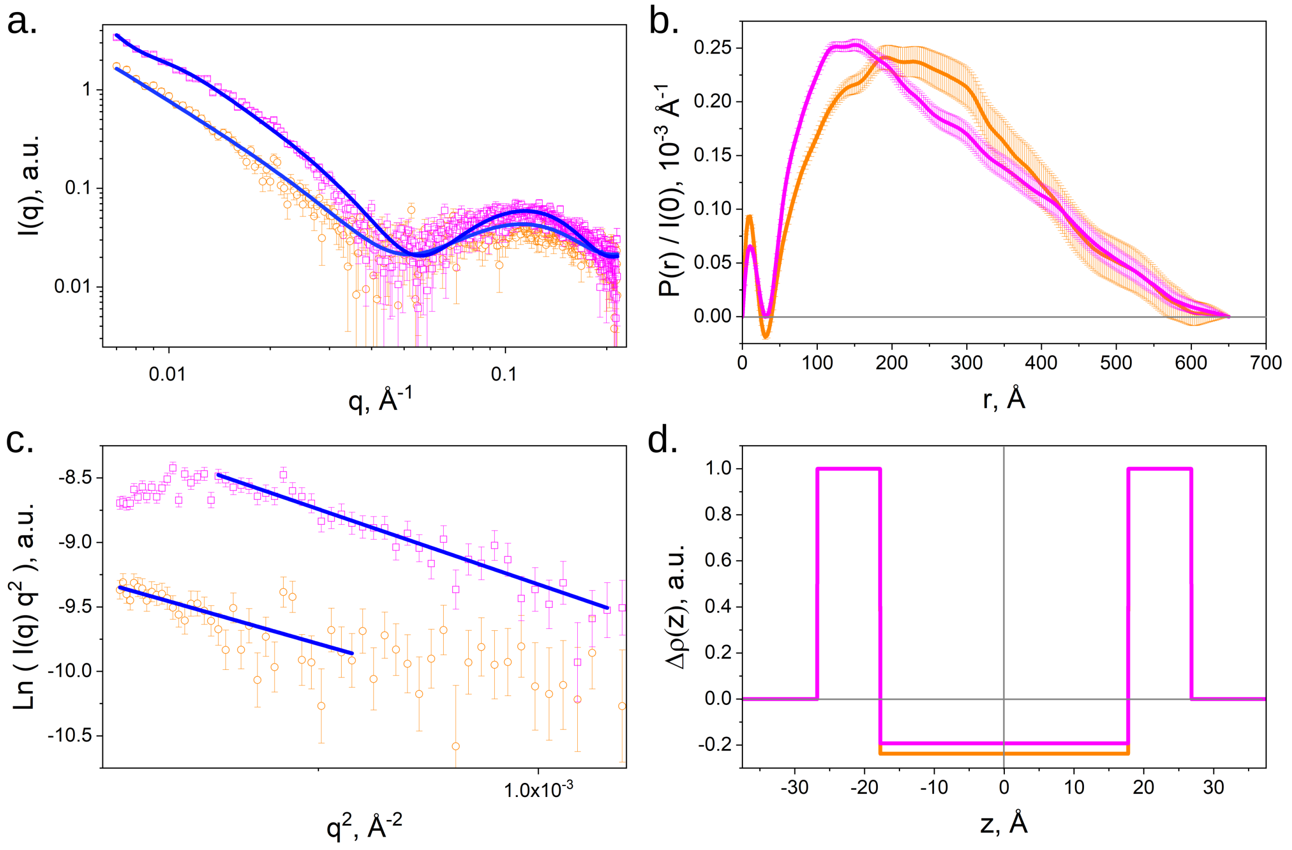

Supplementary Figure 12 | SAXS data for the liposomes prepared from Soybean lecithin polar lipids with the reconstituted SpaR (violet curves) and without the protein (orange curves). **a)** 1D SAXS profiles and the corresponding approximations (blue curves) obtained with the form-factor of ULVs (see material and methods). **b)** Pair distance distribution functions P(r). **c)** Guinier approximations for flat particles. **d)** Normalized profiles of electron density contrast ($\Delta\rho(z)/\Delta\rho_{max}$, $\Delta\rho(z)=\rho(z) - \rho_{buf}$) which correspond to the fitting parameters (see Supplementary Table 2) for the approximations shown in panel **a**.

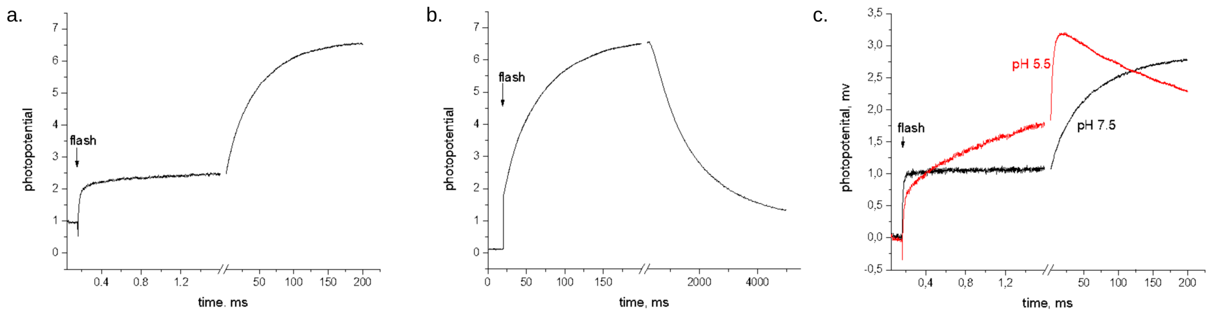

Supplementary Figure 13 | Light-induced membrane potential changes ΔΨ in SpaR proteoliposomes. Membrane potential changes ΔΨ in SpaR proteoliposomes absorbed on the colloidal film after light pulses (designated by arrow and "flash" signature) are shown in: **a)** HEPES-KOH 25 mM pH 7.5 at fast and slow combined scale; **b)** HEPES-KOH 25 mM pH 7.5 at slow scale; **c)** HEPES-KOH 25 mM pH 7.5 (black) and 25 mM MES-KOH pH 5.5 (red) at fast and slow combined scale.

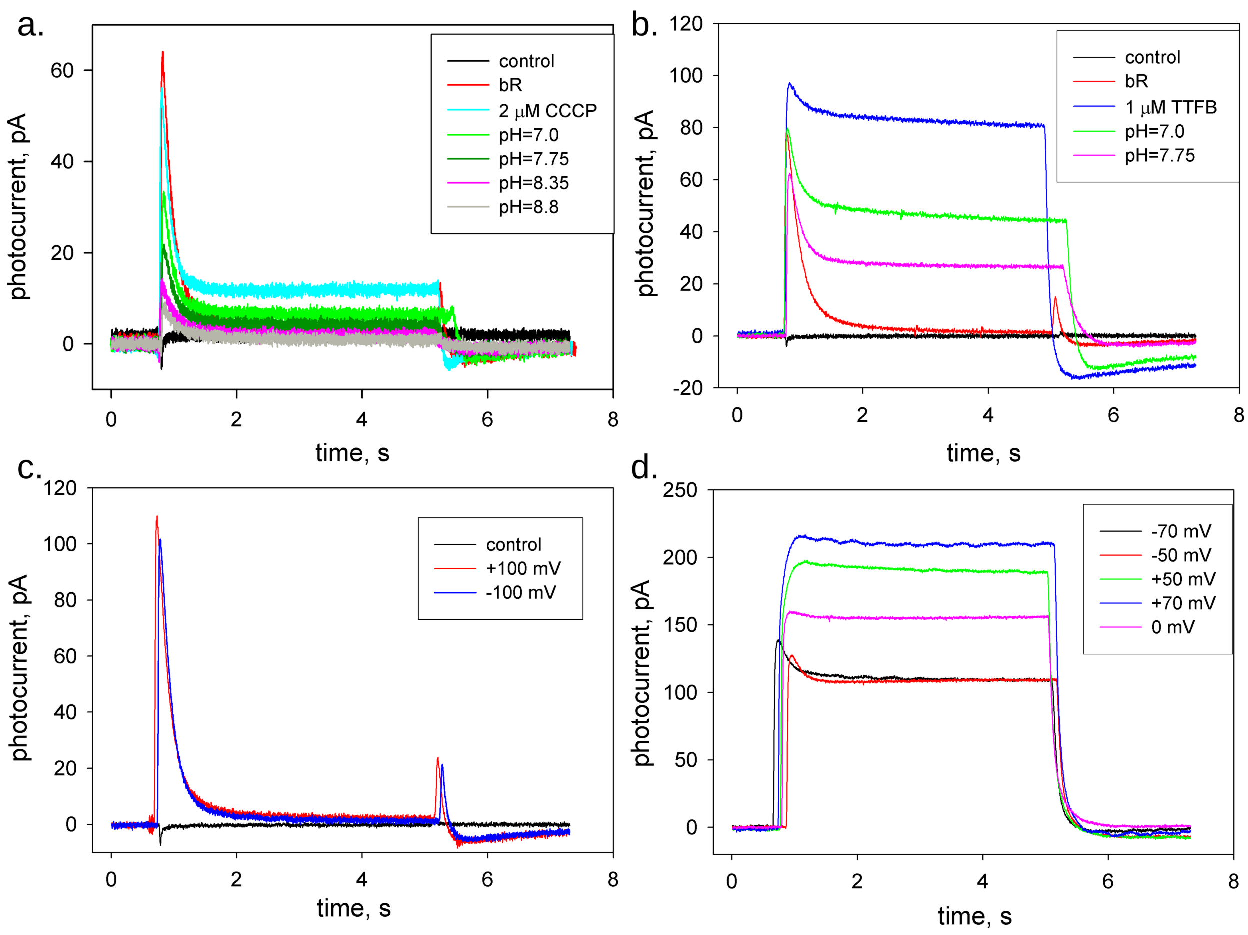

Supplementary Figure 14 | Photocurrents of bR proteoliposomes adsorbed to a planar bilayer lipid membrane (BLM) at different pH and voltages. a) Photocurrent of bR at different pH in the absence (red curve) and in the presence of a protonophore CCCP (other curves). The proteoliposomes adhered to one side of the BLM in a buffer containing 10 mM MES, 10 mM Tris, 100 mM NaCl, pH=6.0. The photocurrents were recorded after an incubation of liposomes during 1 h upon illumination of the white light without a protonophore (red line) and after an addition of 2 μM CCCP (cyan line) at V=0 mV. The pH of the aqueous solution was altered by adding of different aliquots of the Tris solution. **b)** Photocurrent of bR at different pH in the absence (red curve) and in the presence of a protonophore TTFB (other curves). Proteoliposomes adhered to one side of the BLM in a buffer containing 10 mM MES, 10 mM Tris, 100 mM NaCl, pH=6.0. The photocurrents were recorded after incubation of liposomes during 1 h upon illumination of the white light without a protonophore (red line) and after an addition of 0.5 μM TTFB (blue line) at V=0 mV. The pH of the aqueous solution was altered by adding of different aliquots of the Tris solution. **c)** Voltage dependence of the BLM photocurrent of bR proteoliposomes adsorbed to a planar BLM in a buffer containing 10 mM MES, 10 mM Tris, 100 mM NaCl, pH=6.0. **d)** Voltage dependence of the BLM photocurrent of bR proteoliposomes adsorbed to a planar BLM in the presence of 0.5 μM TTFB in a buffer containing 10 mM MES, 10 mM Tris, 100 mM NaCl, pH=6.0. The BLM conductance in the presence of TTFB depended on pH and amounted to 100 nS (pH=6.0), 60 nS (pH=7.0), 19 nS (pH=7.75). The BLM was illuminated by white light.

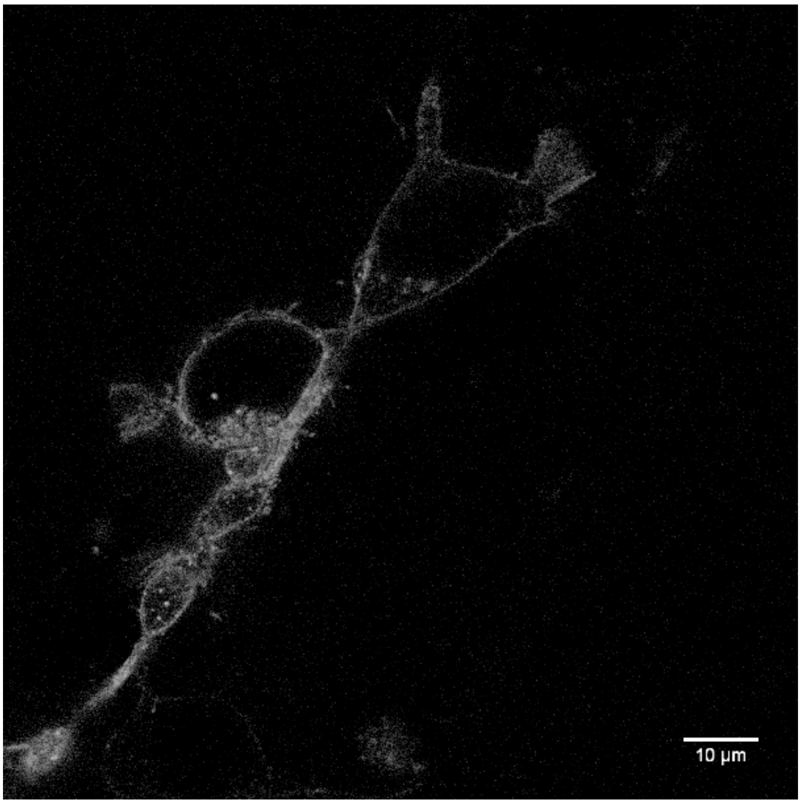

Supplementary Figure 15 | Typical NG108-15 cells expressing SpaR-EYFP in their plasma membrane. Black and white microphotograph shows fluorescence of EYFP protein in in a fused polypeptide chain with SpaR. The scale bar of 10 µm is shown in the lower right corner.

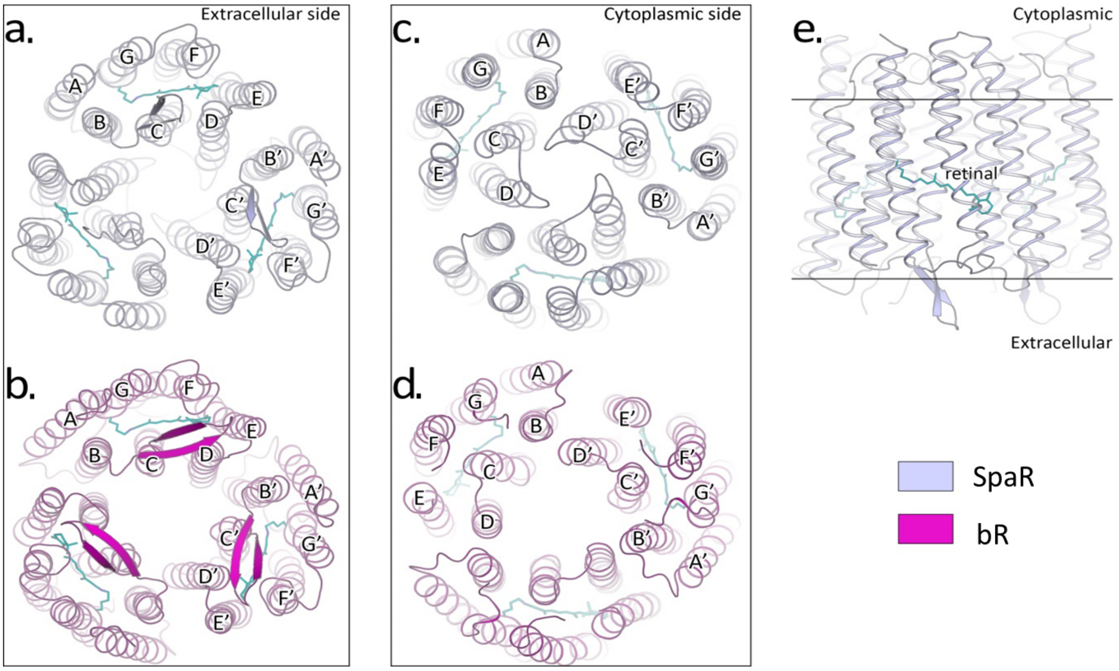

Supplementary Figure 16 | Comparison of the SpaR trimer with bR. a) the view of SpaR from the extracellular side; **b)** the view of bR from the extracellular side; **c)** the view of SpaR from the cytoplasmic side; **d)** the view of bR from the cytoplasmic side. **e)** Side view of SpaR and bR.

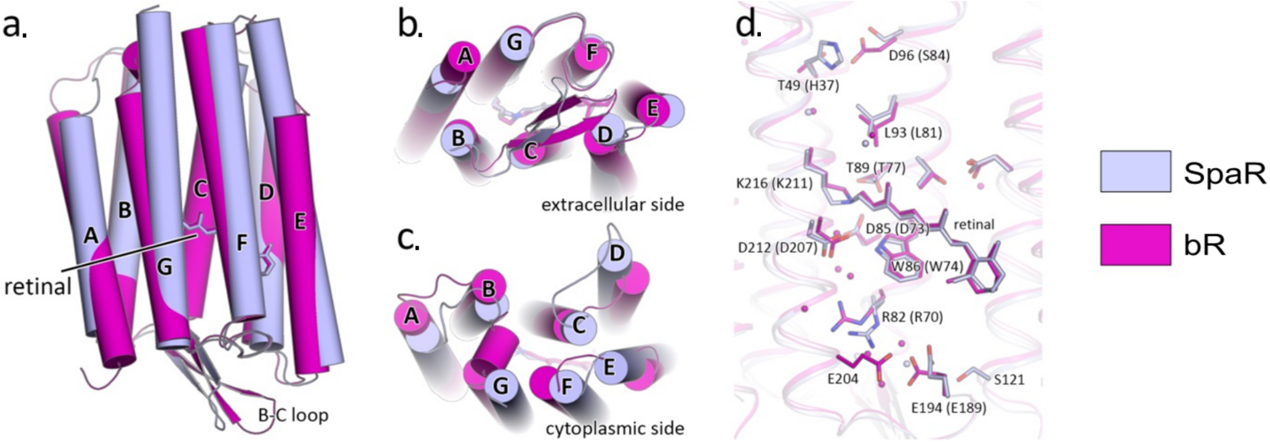

Supplementary Figure 17 | Structural alignment of SpaR with bR. a) Side view. **b)** View from the extracellular side. **c)** View from the cytoplasmic side. The helices are represented by cylinders. **d)** Alignment of key residues of SpaR and BR.

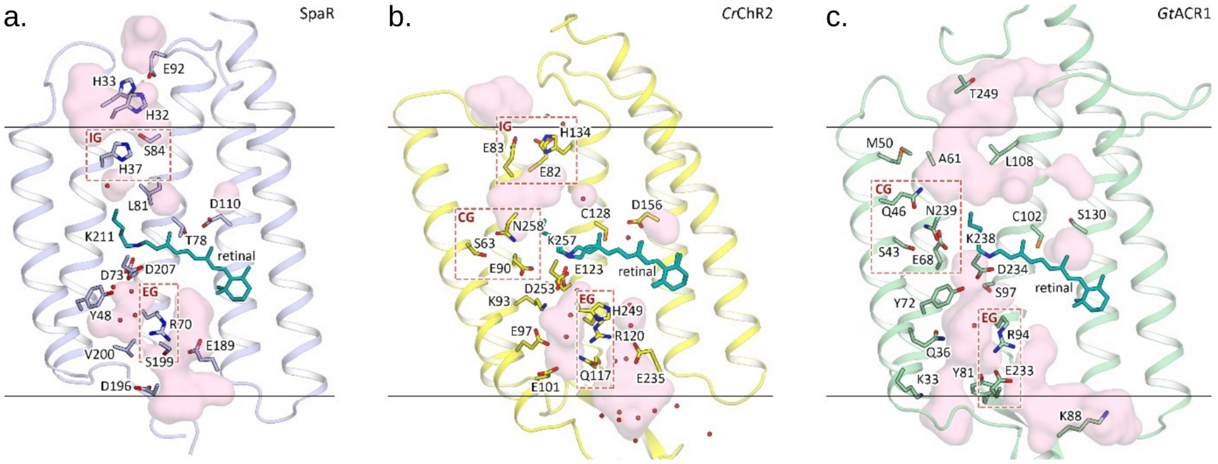

Supplementary Figure 18 | Comparison of overall protomer structures and cavities inside the **a)** SpaR (blue, present work), **b)** CrChR2 (yellow, PDB ID: 6EID) and **c)** GtACR1 (green, PDB ID: 6CSM). Intracellular (IG), central (CG) and extracellular (EG) gates are shown by red dashed rectangles. The cavities are coloured pink. Cofactor retinal is coloured teal. The membrane core boundaries are calculated using PPM server and shown by black lines.

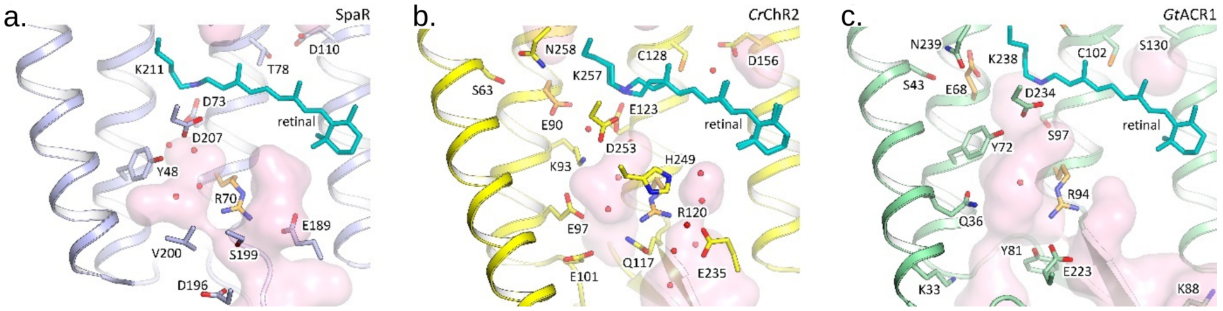

Supplementary Figure 19 | Detailed view of the extracellular part. a) SpaR (blue, present work); **b)** CrChR2 (yellow, PDB ID: 6EID); **c)** GtACR1 (green, PDB ID: 6CSM). Cofactor retinal is coloured teal. The cavities are colored pink. The key charged residues in the gates are colored orange.

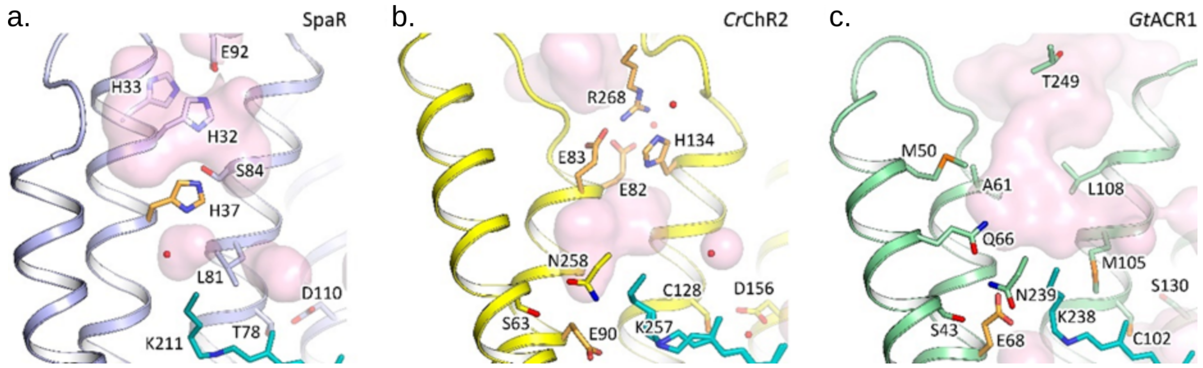

Supplementary Figure 20 | Detailed view of the cytoplasmic part. **a)** SpaR (blue, present work); **b)** CrChR2 (yellow, PDB ID: 6EID); **c)** GtACR1 (green, PDB ID: 6CSM). Cofactor retinal is coloured teal. The cavities are coloured pink. The key charged residues of the gates are coloured orange.

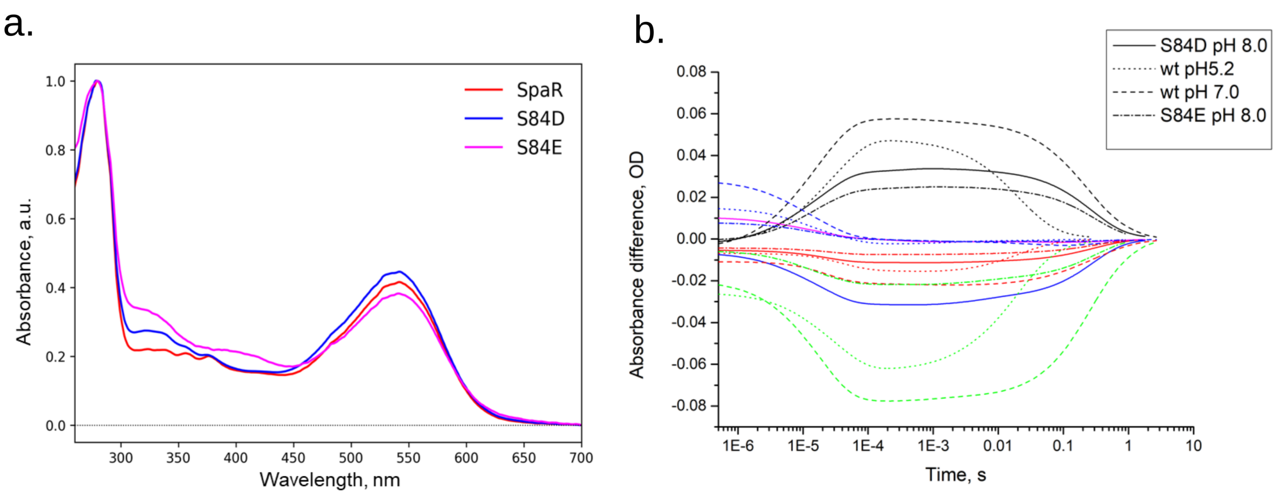

Supplementary Figure 21 | Spectral comparison of DDM-solubilized S84D, S84E mutants and wild-type SpaR. a) The absorption spectra of DDM-solubilized S84D, S84E mutants of SpaR in comparison with those of wild-type SpaR. **b)** Kinetics of the light-induced absorption changes in the photocycle of DDM-solubilized S84D, S84E mutants of SpaR at pH 8.0 in comparison with SpaR at pH 5.2 and pH 7.0.

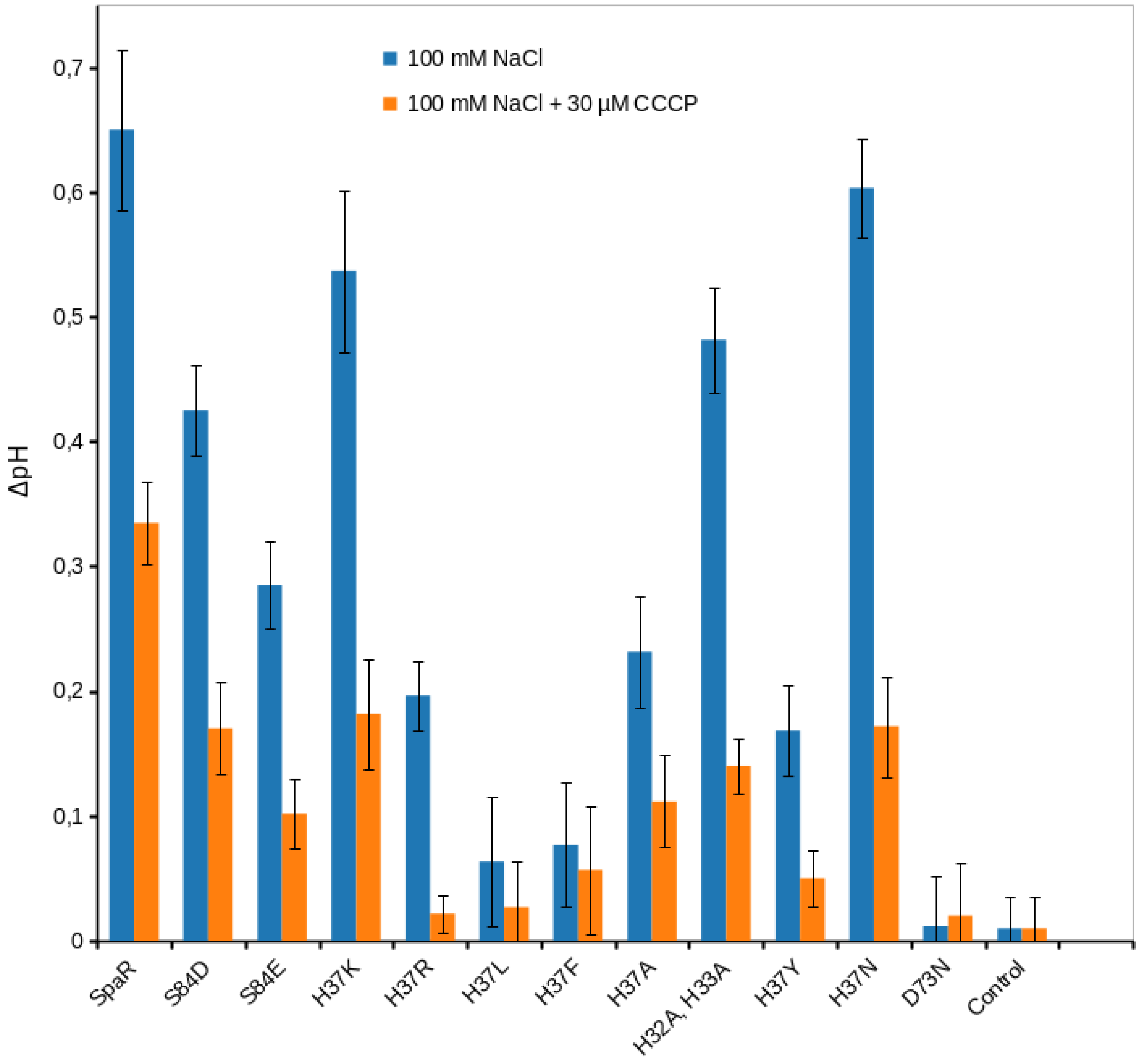

Supplementary Figure 22 | Pumping activity of SpaR and its mutants expressed in *E. coli*. The *E. coli* cells expressing the SpaR or it's mutant variants were used in suspension in unbuffered solution of 100 mM NaCl without and with thee addition of 30 µM CCCP. The bars represent the maximum changes of pH (modulo value) in suspension reached upon illumination in 6 independent experiments (standard deviations are shown). Data was obtained from ΔpH(time) plots like shown at Supplementary Figure 11.

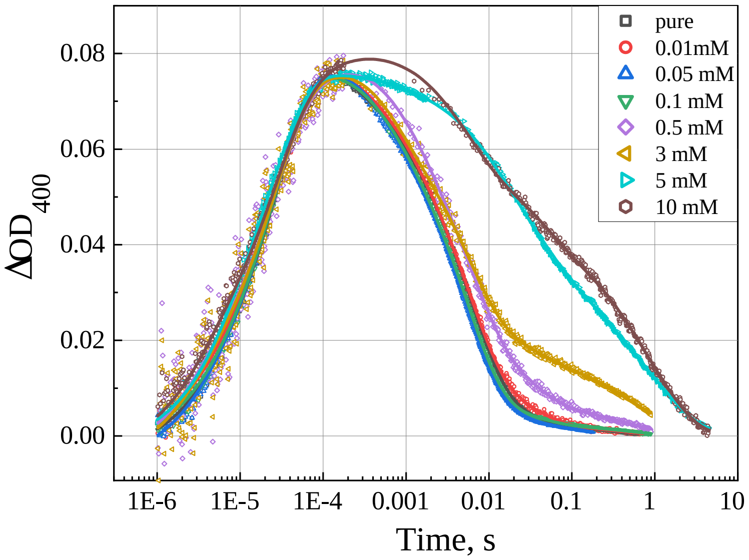

Supplementary Figure 23 | Traces of the transient absorption changes at 400 nm after photoexcitation of SpaR at different Zn^{2+} concentrations. Traces of the transient absorption changes at 400 nm in time after the photoexcitation at different concentrations of Zn^{2+} (0.0, 0.01, 0.05, 0.1, 0.5, 3.0, 5.0, 10.0 mM) are shown by different colours according the legend.

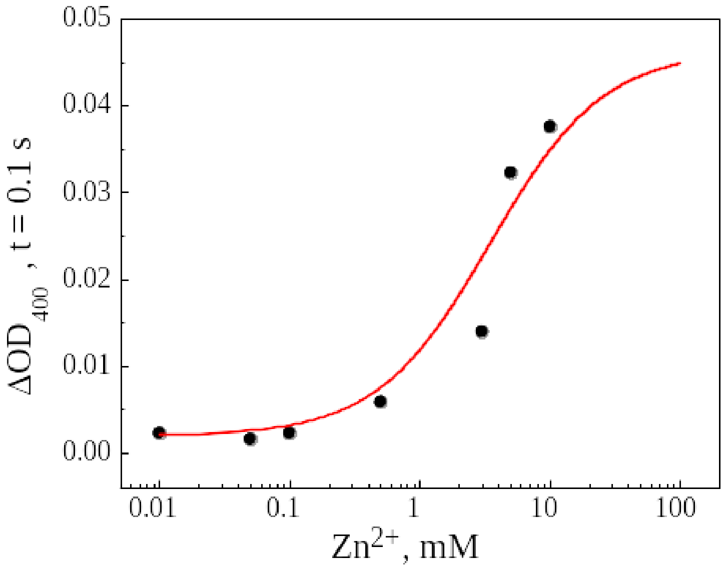

Supplementary Figure 24 | Zn^{2+} concentration dependence of amplitude of the absorption change of the slowest intermediate of the SpaR's as recorded at time=0.1s (Supplementary Figure 23). The data points (black cycles) were fitted by the hyperbolic function $\Delta OD = A_0 + A_1 \frac{[Zn^{2+}]}{K_d + [Zn^{2+}]}$. The solid red curve depicts the data theoretical fit giving *Kd*=3.8±0.8 mM.

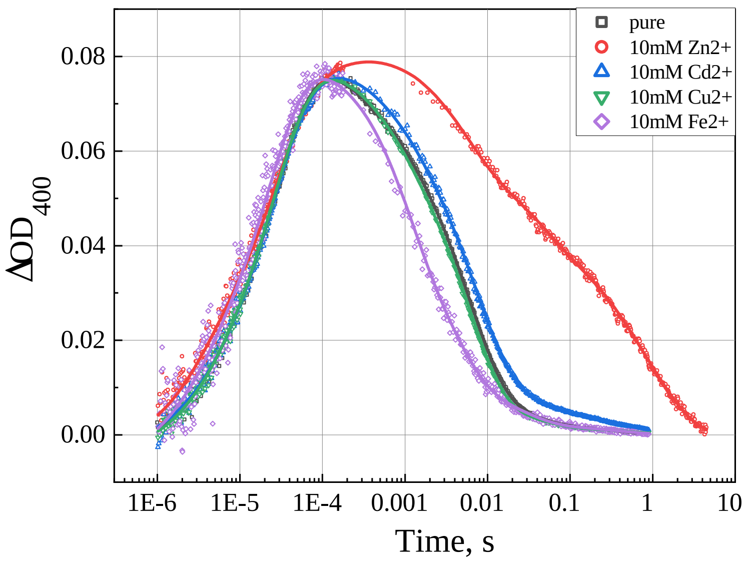

Supplementary Figure 25 | Time traces of the absorption changes of SpaR at 400 nm measured at 10 mM of different divalent cations. Traces of the transient absorption changes at 400 nm in time after the photoexcitation at 10 mM of different divalent ions (Zn^{2+}, Cd^{2+}, Cu^{2+}, Fe^{2+}) are shown by different colours according the legend.

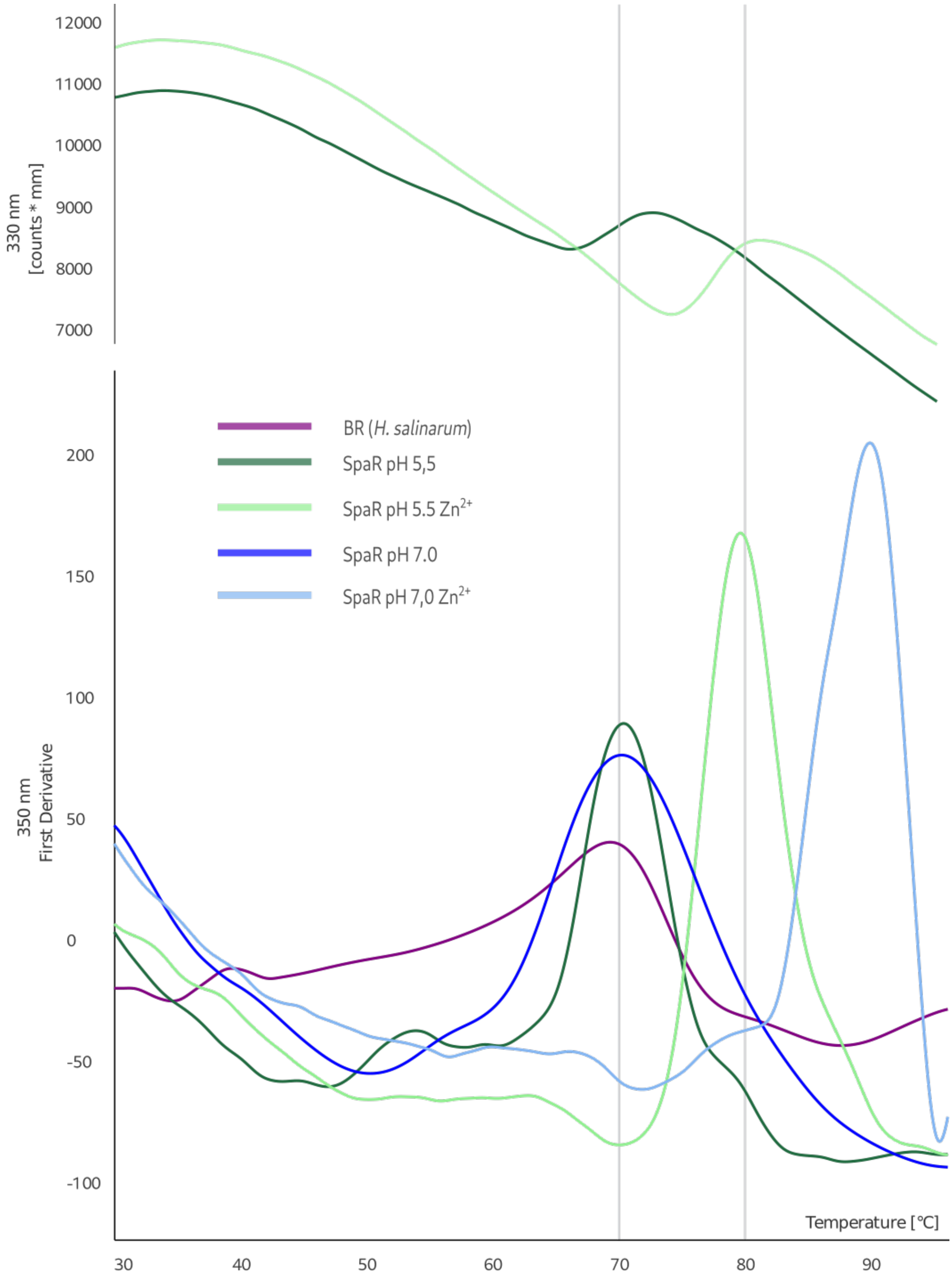

Supplementary Figure 26 | Melting temperature of SpaR in the presence and the absence of Zn^{2+} at pH5.5 and pH7.0. At 10 mM Zn^{2+} SpaR at pH 7.0 shows much higher (up to 10 degrees) temperature-induced denaturation than at pH 5.5. Protein melting temperatures were determined as maxima of the first derivatives (lower part of the combined graph) of the 330 nm SpaR intrinsic fluorescence (upper part of the combined graph) during slow heating rate of 0.5°C per minute (abscissa axis) by the nanoDSF Prometheus Panta instrument (Nanotemper, Germany).

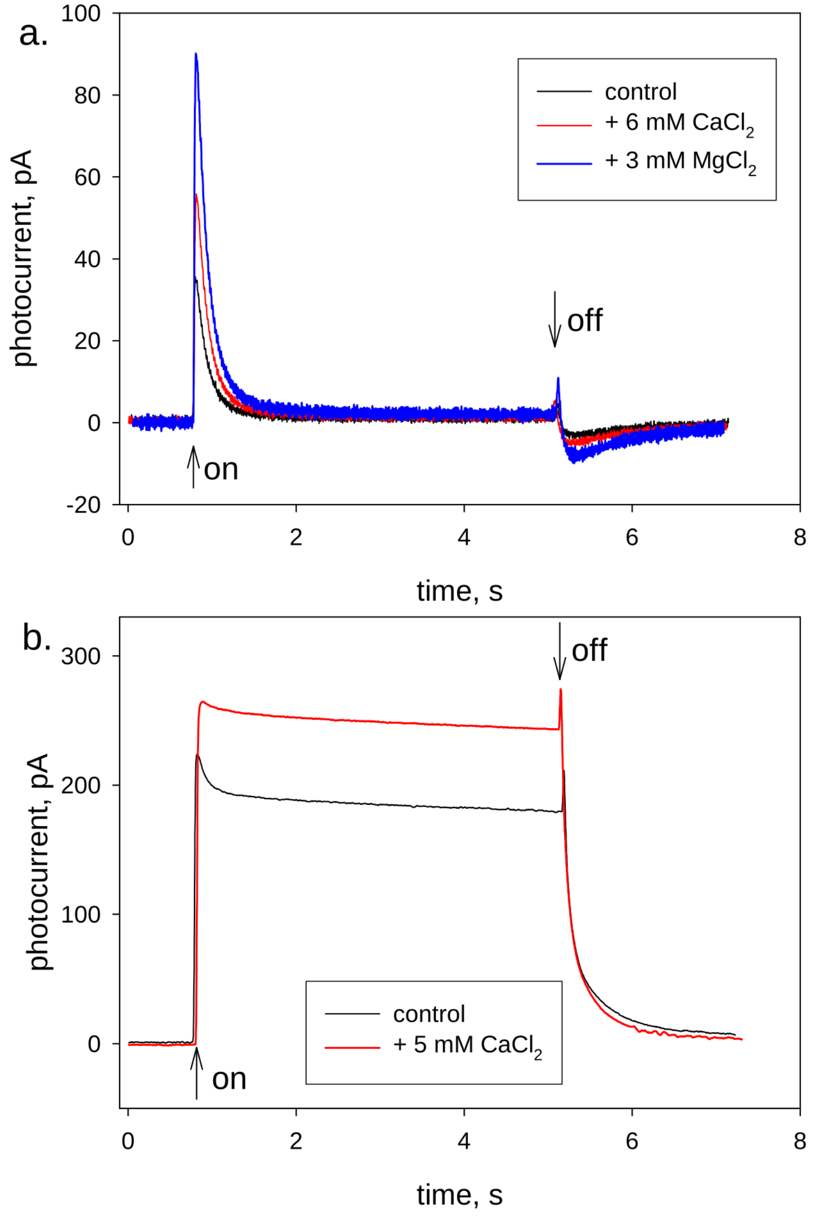

Supplementary Figure 27 | Effect of calcium and magnesium ions on photocurrents of proteoliposomes with SpaR adsorbed to a planar bilayer lipid membrane (BLM). Dependence of photocurrent on time and illumination (started at the time moment shown by the arrow and "on") in the **a)** absence of a protonophore and **b)** in the presence of 0.5 μM TTFB in buffer solution 5 mM MES, 5 mM Tris, 100 mM NaCl, pH 6.0.

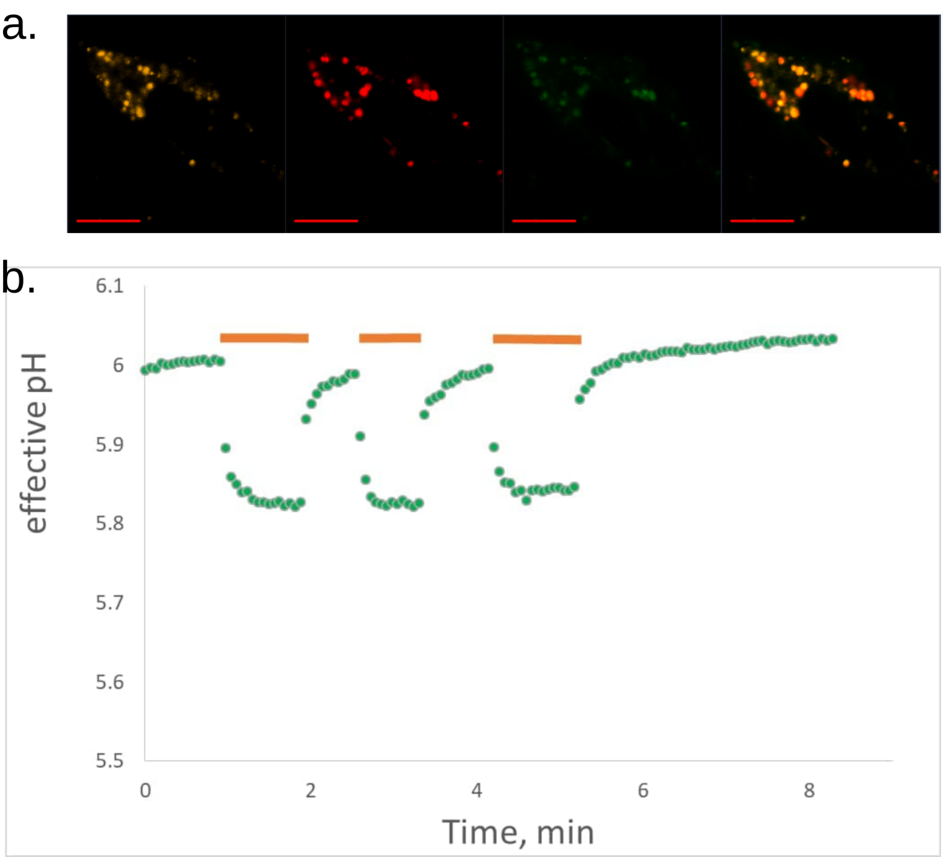

Supplementary Figure 28 | SpaR optogenetically changes pH in lysosomes. a) Hybrid protein CD63-pHluorin-SPaR-mKate2 was expressed in HEK293T cells and localizes with LysoTracker Red DND-99. mKate2 (red), pHluorin (green) and LysoTracker (orange) reveal all, alkaline and acidic organelles correspondingly. The HEK293T cells were untreated. Scale bar 10 um. **b)** Green fluorescent protein pHluorin, bleached under acidification, demonstrates a SpaR-performed outward proton flux (into lysosomes) under 590nm LED illumination. The fluorescence is measured at regions of lysosomes. The time-intervals of illumination with 590nm light are highlighted by orange lines. The HEK293T cell were treated with 50 nM Bafilomycin 12h for full V-ATPase inhibition and lysosomal (a.u., arbitrary units).
