# Supplementary material for: Mirror proteorhodopsins
Source: Commun Chem. 2023 May 2;6:88. doi: 10.1038/s42004-023-00884-8 (PMC10154332; doi:10.1038/s42004-023-00884-8)
Supplement: Supplementary file 2 — Description of Additional Supplementary File [file 42004_2023_884_MOESM2_ESM.pdf]

Description of Additional Supplementary Files

File name: Supplementary Data 1

Description: 8ANQ.cif file.
